# Burden of Non‑Communicable Diseases Among Children and Adolescents in the Asia‑Pacific Region, 1990–2021: Analysis for the Global Burden of Diseases Study 2021

**DOI:** 10.5334/aogh.4891

**Published:** 2025-12-17

**Authors:** Dandan Bai, Wu Yan, Jinlong Chen, Sabitina Mrisho Mzava, Francis Manyori Bigambo, Xu Wang, Yanqun Sun

**Affiliations:** 1 Department of Pediatrics, Affiliated Hospital of Nantong University, Nantong, Jiangsu Province, China; 2 Clinical Medical Research Center, Children’s Hospital of Nanjing Medical University, Nanjing, 210008, China; 3 Department of Cardiology, Children’s Hospital of Nanjing Medical University, Nanjing, 210008, China; 4 Nanjing Medical University, Nanjing, 210008, China

**Keywords:** non‑communicable diseases, death, disability‑adjusted life years, years of life lost, years lived with disability, children and adolescents, Asia‑Pacific region

## Abstract

*Background:* Non-communicable diseases (NCDs) have surpassed infectious diseases as the leading cause of disability and mortality globally. However, the burden of NCDs among children and adolescents in the Asia-Pacific region remains underexplored. This study evaluates changes in NCD burden among individuals aged 0–19 years in the Asia-Pacific region from 1990 to 2021.

*Methods:* Using data from the Global Burden of Diseases, Injuries, and Risk Factors (GBD) 2021 study, we estimated deaths, disability-adjusted life years (DALYs), years of life lost (YLLs), and years lived with disability (YLDs) with 95% uncertainty intervals (UIs). We analyzed changes in disease burden by age, sex, location, and socio-demographic index (SDI) between 1990 and 2021.

*Results:* In 2021, NCDs accounted for a YLD rate of 56488.99 (95% UI: 40849.26–75780.79) and a death rate of 356.45 (95% UI: 280.27–433.27) per 100,000 population among children and adolescents aged 0–19 years. Other NCDs were the leading cause of death (167.08 [95% UI: 116.63–216.90]), YLLs (14584.60 [95% UI: 10126.88–18,962.14]), and DALYs (20327.20 [95% UI: 14815.25–26238.15]) per 100,000, while mental disorders led in YLDs. Death rates were higher in males, but DALYs were higher in females. From 1990 to 2021, NCD death rates decreased by 44.63% (from 643.71 [95% UI: 508.48–758.86] to 356.45 [95% UI: 280.27–433.27]) and YLLs by 45.49%. However, mental disorder-related deaths, YLLs, and DALYs increased by 29.26%, 29.16%, and 10.07%, respectively. Lower SDI countries reported higher NCD burdens, particularly for other NCDs.

*Conclusions:* While the NCD burden among children and adolescents in the Asia-Pacific region decreased significantly from 1990 to 2021, the rising burden of mental disorders is a critical public health concern.

## Introduction

Non‑communicable diseases (NCDs) are long‑lasting and slow‑progressing conditions that cannot be transmitted from person to person [1, 2]. Behaviors are the main driving factors for NCDs, which typically originate in childhood and adolescence, including unhealthy eating habits, physical inactivity, tobacco use, and excessive alcohol consumption. These behaviors can adversely affect the health of children and adolescents, leading to negative health outcomes in adulthood [1, 2].

Despite the significant advancements in effective and safe preventive strategies on a global scale,NCDs, including cardiovascular diseases, neoplasms, diabetes, and chronic respiratory diseases, remain the primary causes of morbidity and mortality [3]. Approximately 41 million individuals succumb to NCDs annually, which constitutes 74% of all deaths worldwide [4]. An estimated 4.7 billion out of the total global population of 8 billion reside in the Asia‑Pacific region, with projections indicating an increase to 5.2 billion by the year 2050 [4]. In the Asia‑Pacific region, there is a drastic increase in NCDs and related deaths, specifically in the Southeast Asian region, where the burden of NCDs is severe [3]. The burden of both communicable diseases and NCDs creates a greater challenge to the healthcare system of Southeast Asia, as the healthcare system has been prepared to focus on managing communicable diseases and acute care and is not well designed to deal with the needs of chronic care like NCDs [3, 5]. Additionally, research has documented that children and adolescents in the Asia‑Pacific region are not adequately physically active compared to other parts of the world [6] and only 6% achieved the WHO’s target of 60 min of moderate‑ to vigorous‑intensity physical activity (MVPA) each day [7].

Previous studies conducted in some of the countries in the Asia‑Pacific region reported the burden of NCDs among children and adolescents in different contexts. For instance, the Global Burden of Disease (GBD) study 2019, focusing on China only, found that NCDs contribute to 50.30% of all‑cause DALYs in children and adolescents of 0–19 years old [8], while mental disorders were the biggest contributor to DALYs [1]. Another GBD study in 2019 focusing on Southeast Asia and Western Pacific regions found that NCDs contributed to 27.3% of total deaths in Southeast Asia and 34.6% in the Western Pacific region as well as 49.8% of total DALYs in South Asia and 65.1% in Western Pacific among adolescents aged 10–24 years, with neoplasms, cardiovascular diseases, and mental disorders being the leading causes of NCDs across 42 countries [9]. A nationally representative study conducted in Malaysia showed that the prevalence of depression was 26.9% among children and adolescents aged 3–17 years, with a higher prevalence in females than males, and the prevalence increased with age [10].

To our knowledge, no comprehensive study has been published to assess the burden of NCDs across countries in the Asia‑Pacific region. Based on GBD 2021 data, the current study thoroughly assesses the burden of NCDs among children and adolescents aged 0–19 in the Asia‑Pacific region from 1990 to 2021. The insights derived from this research will assist nations in identifying critical intervention priorities and establishing a baseline for evaluating the effectiveness of long‑term programs and policies.

## Method Details

This study estimates the burden of NCDs among children and adolescents in the Asia‑Pacific region using the GBD 2021 data. GBD 2021 generated estimates for 204 countries and territories grouped into 21 regions and seven super‑regions. The study estimated the disability‑adjusted life years (DALYs), years of life lost (YLLs), years lived with disability (YLDs), incidence, prevalence, and healthy life expectancy (HALE) for 371 diseases and injuries. The estimation of DALYs was based on the information from 100,983 data sources, while that of YLLs was based on the data sources discovered in the previous cycles, along with 9248 newly identified sources. The University of Washington Institutional Review Board committee approved the GBD study 2021 (STUDY00009060). Informed consent was waived because of the use of anonymized data. This paper was produced according to the GBD protocol. The reporting followed the Guidelines for Accurate and Transparent Health Estimates Reporting Statement (GATHER) [11] (see supplemental research checklist). The detailed methods are presented in previous studies [12, 13] and supplemental methods.

## Data Analysis

In this study, we highlighted all three level 1 causes of disease burdens to provide a general understanding of the causes: communicable, maternal, neonatal, and nutritional status; NCDs; and injuries. We then reported in detail the level 2 and 3 causes of NCDs, which are the main focus of our study (Table S1).

We produced the estimates for the Asia‑Pacific region, including seven GBD regions: Australasia, Central Asia, East Asia, high‑income Asia‑Pacific, Oceania, South Asia, and Southeast Asia, which comprise 54 countries (Table S2). The estimates were computed from 1990 to 2021, stratified by sex and age groups. Death, DALYs, YLLs, and YLDs were presented as rates per 100,000 population. We presented these metrics for all‑cause, level 1, NCD level 2, and exclusive NCD level 3; sex (female, male, both sexes); age groups (0–6 days, 7–27 days, 1–5 months, 6–11 months, 1 year, 2–4 years, 5–9 years, 10–14 years, 0–14 years, 15–19 years, and 0–19 years [after merging 0–14 and 15–19 years]); location (54 countries in the Asia‑Pacific region); and trend from 1990 to 2021, and then we computed the percentage change between 1990 and 2021 in 0–19 years old. The estimates produced in the GBD 2021 were presented with 95% uncertainty intervals (UIs). We also presented each country’s social demographic index (SDI) in the Asia‑Pacific region.

## Results

### Death

In 2021, the all‑cause death rate among children and adolescents aged 0–19 years in the Asia‑Pacific region was 1585.91 (95% UI: 1375.71, 1832.18) per 100,000 population (Figures S1 and S2). The death rate due to NCDs was 356.45 (95% UI: 280.27, 433.27) per 100,000 population (Table S3). Other NCDs were the leading level 2 cause of death (167.08 [116.63, 216.90] per 100,000 population), followed by neoplasms (56.88 [47.40, 67.50] per 100,000 population) and cardiovascular disease (48.60 [39.68, 59.06] per 100,000 population). The leading level 3 causes of death were congenital birth defects (145.77 [98.49, 192.40] per 100,000 population) and leukemia (19.62 [14.63, 25.13] per 100,000 population) (Table S4).

In 2021, NCDs were the second level 1 cause of death in females and males of all age groups, except for females aged 15–19 years, where it was the first. In both sexes, NCDs were the second level 1 cause of death across all age groups, except for 10–14 years, where NCDs were the third (Table S3). Age and sex differences in death rates due to NCDs are presented in Figure S3.

**Figure 1:**
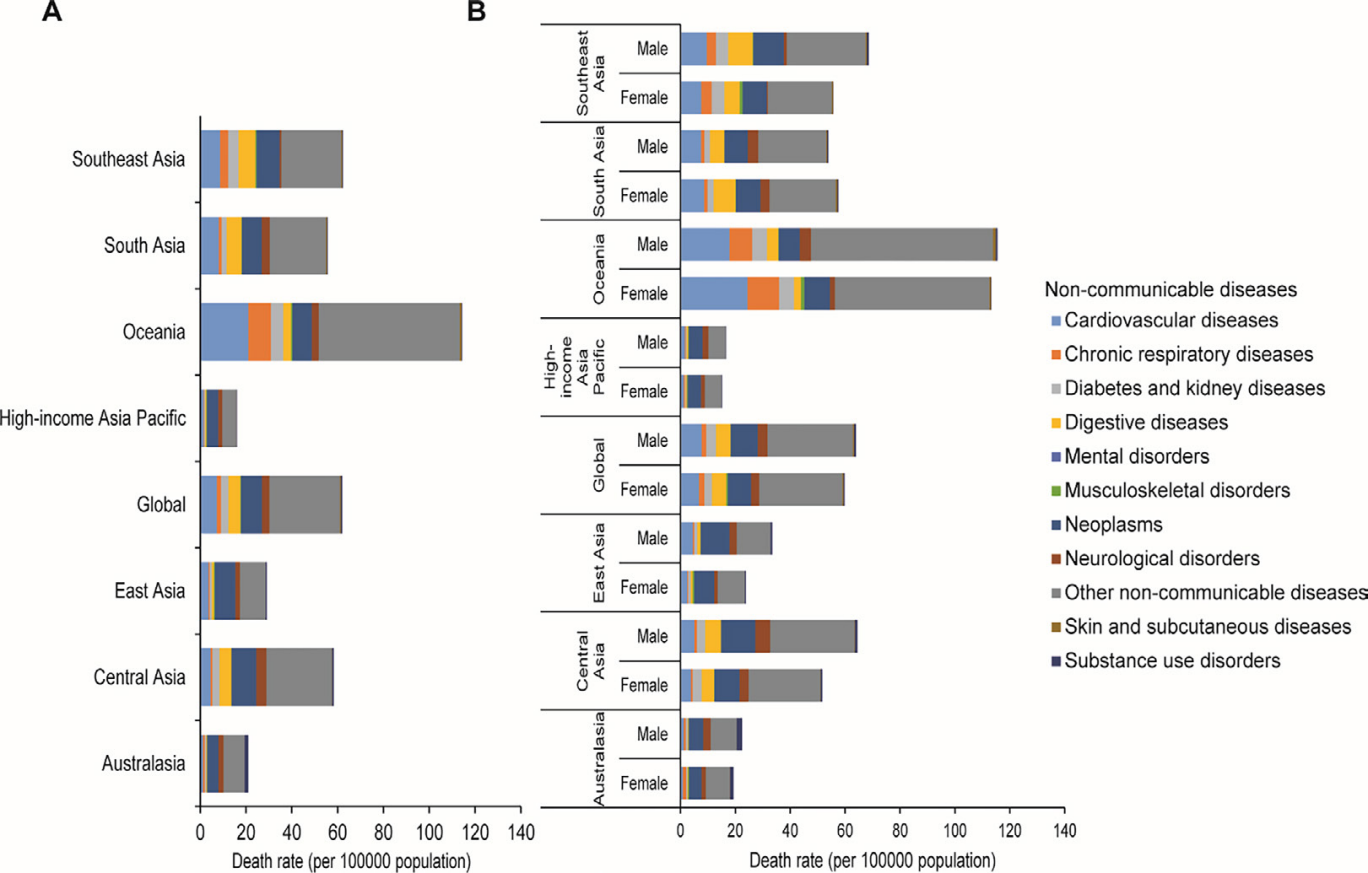
Death rate per 100,000 population due to level 2 non‑communicable diseases among children and adolescents aged 0–19 years by region in 2021. **(A)** Both sexes. **(B)** Females and Males.

**Figure 2:**
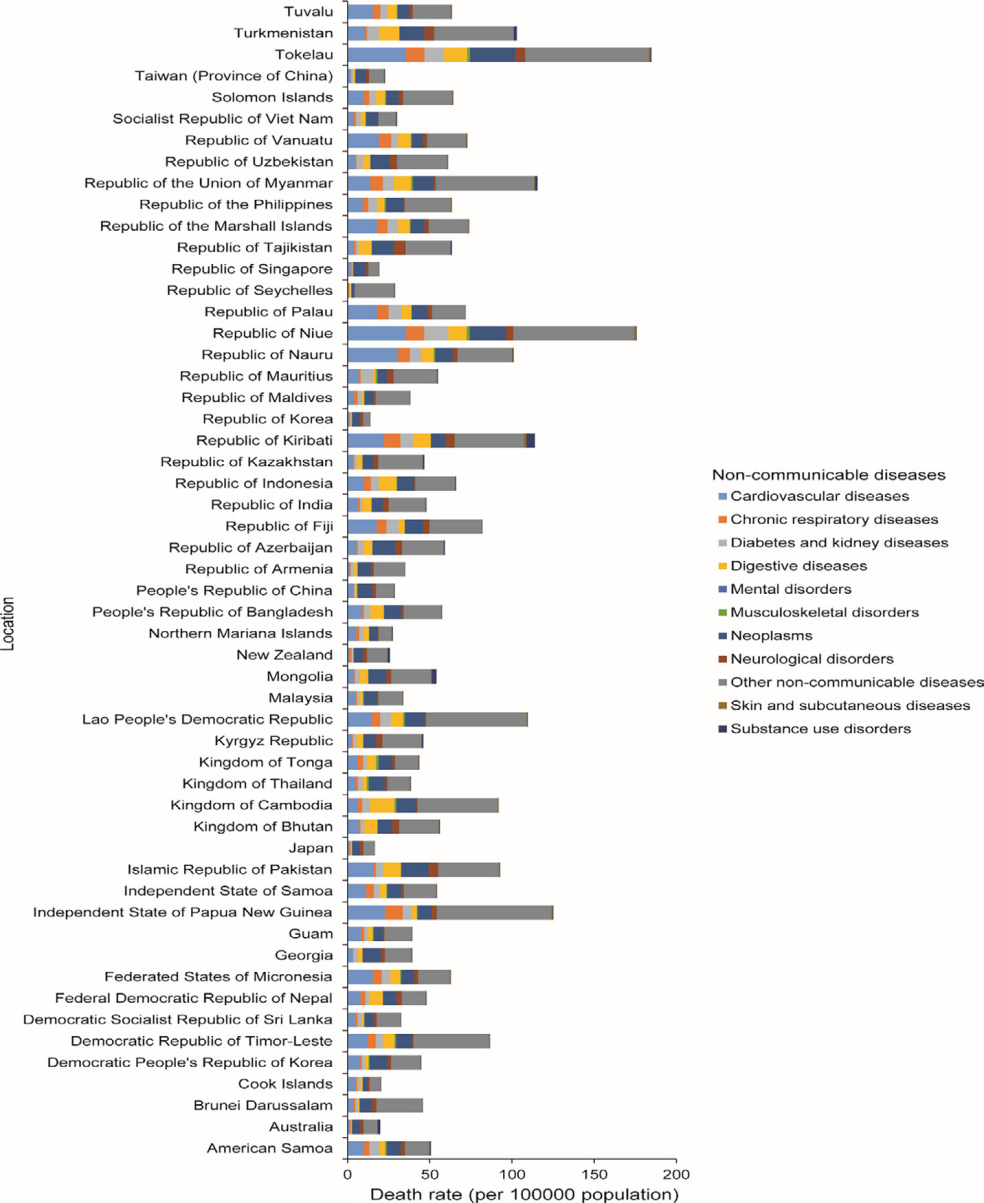
Death rate per 100,000 population due to level 2 non‑communicable diseases among children and adolescents aged 0–19 years in both sexes, by country in 2021.

The highest and lowest death rates due to level 2 NCDs were observed in the Oceania and high‑income Asia‑Pacific regions, respectively (Figure 1A). Across the Asia‑Pacific region, males had a higher death rate than females (Figure 1B). Tokelau has the highest death rate, followed by Niue. The Republic of Korea and Japan had the lowest death rates. Other NCDs were the leading level 2 cause of death (Figure 2).

From 1990 to 2021, the death rate due to NCDs decreased significantly from 643.71 (508.48, 758.86) to 356.45 (280.27, 433.27) per 100,000 population, equivalent to a 44.63% decrease. For level 2 causes, the highest decline in the death rate due to NCDs was observed in digestive disorders (–59.35 [–59.23 to –59.45]) and other NCDs (–48.12 [–47.35, −45.62]), whereas mental disorders revealed the highest increase (29.26 [24.45 to 35.97] per 100,000 population), particularly eating disorders, which is level 3 cause of NCDs (Table S4, Figure S4).

### Years of life lost

In 2021, the all‑cause YLL rate among children and adolescents was 132815.83 (95% UI: 114886.60, 153898.36) per 100,000 population (Figure S5). NCDs were the second level 1 cause of YLLs (29159.23 [22698.30, 35630.45] per 100000 population) (Table S3). In the NCDs, other NCDs were the leading level 2 cause of YLLs (14584.60 [10126.88, 18962.14] per 100,000 population), followed by neoplasms (4378.01 [3643.71, 5208.37] per 100,000 population) and cardiovascular diseases (3728.41 [3038.92, 4538.05] per 100,000 population). The top three level 2 causes of NCDs were congenital birth defects, leukemia, and stroke (Table S5).

**Figure 3:**
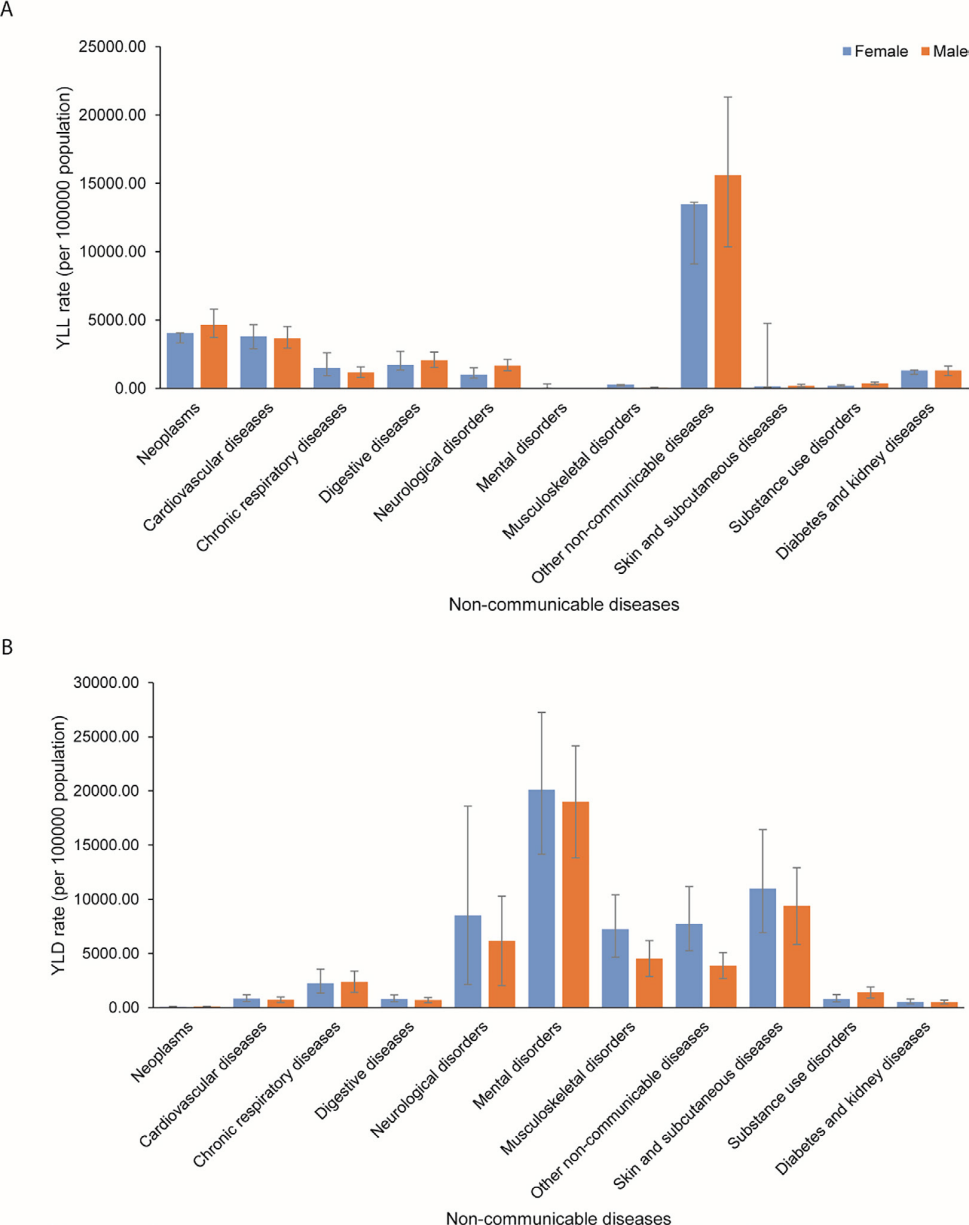
YLL **(A)** and YLD **(B)** rates per 100,000 population due to level 2 non‑communicable diseases among children and adolescents aged 0–19 years in the Asia‑Pacific region by sex in 2021.

In 2021, the all‑cause YLL rate per 100,000 population was higher in males than in females. The greatest difference was observed in the age groups 0–6 days and 15–19 years (Figure S5). NCDs were the second leading cause of YLLs in females and males in all age groups (Table S3). Sex and age group differences for YLLs due to level 2 NCDs are shown in Figure 3A and Figure S6. Substantial sex differences in YLL rate per 100,000 population due to level 3 NCDs were observed in congenital birth defects, leukemia, and idiopathic epilepsy (Figure S7).

In 2021, the highest and lowest burdens of YLL rates due to NCDs were observed in the Republic of Korea and Tokelau, respectively. Other NCDs were the leading level 2 cause of YLL across all countries in the Asia‑Pacific region, except in the Republic of Korea, where it was neoplasms (Figure S8).

Between 1990 and 2021, the YLL rate due to NCDs decreased across the region; the only NCD level 2 that increased the YLL rate by 29.16% was mental disorders, particularly eating disorders (Table S5 and Figure S9).

### Years lived with disability

In 2021, the all‑cause YLD rate among children and adolescents was 76230.23 (95% UI: 55703.21, 101450.08) per 100,000 population (Figure S10). The leading level 1 cause of YLD was NCDs (56488.99 [95% UI: 40849.26, 75780.79] per 100,000 population) (Table S3). Mental disorders were the leading level 2 NCD causes of YLD (19537.66 [14033.65, 26085.25] per 100,000 population), while the top three level 3 causes of YLD were anxiety, headache, and depressive disorders (Table S4).

The all‑cause YLD rates were higher in females than in males (Figure S10). The leading level 1 cause of YLD was NCDs, and the greater burden was observed in the age group 15–19 years (Table S3). For both groups, mental disorders were the leading level 2 cause of YLDs (Figure S11). Sex and age group differences for YLDs due to level 2 NCDs are shown in Figure 3B and Figure S11. The substantial differences in YLD rates per 100,000 population due to level 3 causes of NCDs among females and males aged 0–19 years were present in depressive, anxiety, and headache disorders (Figure S12).

In 2021, the YLD rates due to NCDs were highest in New Zealand and Australia and lowest in Azerbaijan and China, with mental disorders being the leading level 2 cause of YLD rates across the Asia‑Pacific region (Figure S13).

There was a significant change in all‑cause YLD rates among children and adolescents from 82756.01 (95% UI: 60542.71, 110113.84) to 76230.23 (95% UI: 55703.20773, 101450.01) between 1990 and 2021 (Figure S10). The greatest increase in the YLD rates due to level 2 NCDs was observed in diabetes and kidney diseases from 392.51 (255.85, 575.05) to 526.75 (344.08, 783.91) between 1990 and 2021, equivalent to a 34.20% increase (Table S5 and Figure S14).

### Disability‑adjusted life years

The all‑cause DALY rate per 100,000 population among children and adolescents was 209046.06 (95% UI: 176872.97, 246743.48) (Figure S15). NCDs were the second level 2 cause of the DALY rate (Table S3). Even though other NCDs were the leading cause of DALY rates, mental disorders increased the DALY rate by 10.07% between 1990 and 2021. The top three causes of DALY rates due to level 3 NCDs were anxiety, headache, and depressive disorders (Table S4).

In 2021, the all‑cause DALY rate per population decreased across all age groups and was higher in males than in females (Figure S15). NCDs were the second level 1 cause of DALY rates across all age groups and sexes, except for the age groups 5–9 years, 10–14 years, and 15–19 years, where the leading level 1 cause of DALYs was NCDs (Table S3). Sex and age group differences for DALY due to level 2 NCDs are presented in Figure S16. The leading level 3 causes of NCDs were congenital birth defects, anxiety, headache, and depressive disorders (Figure S17).

**Figure 4:**
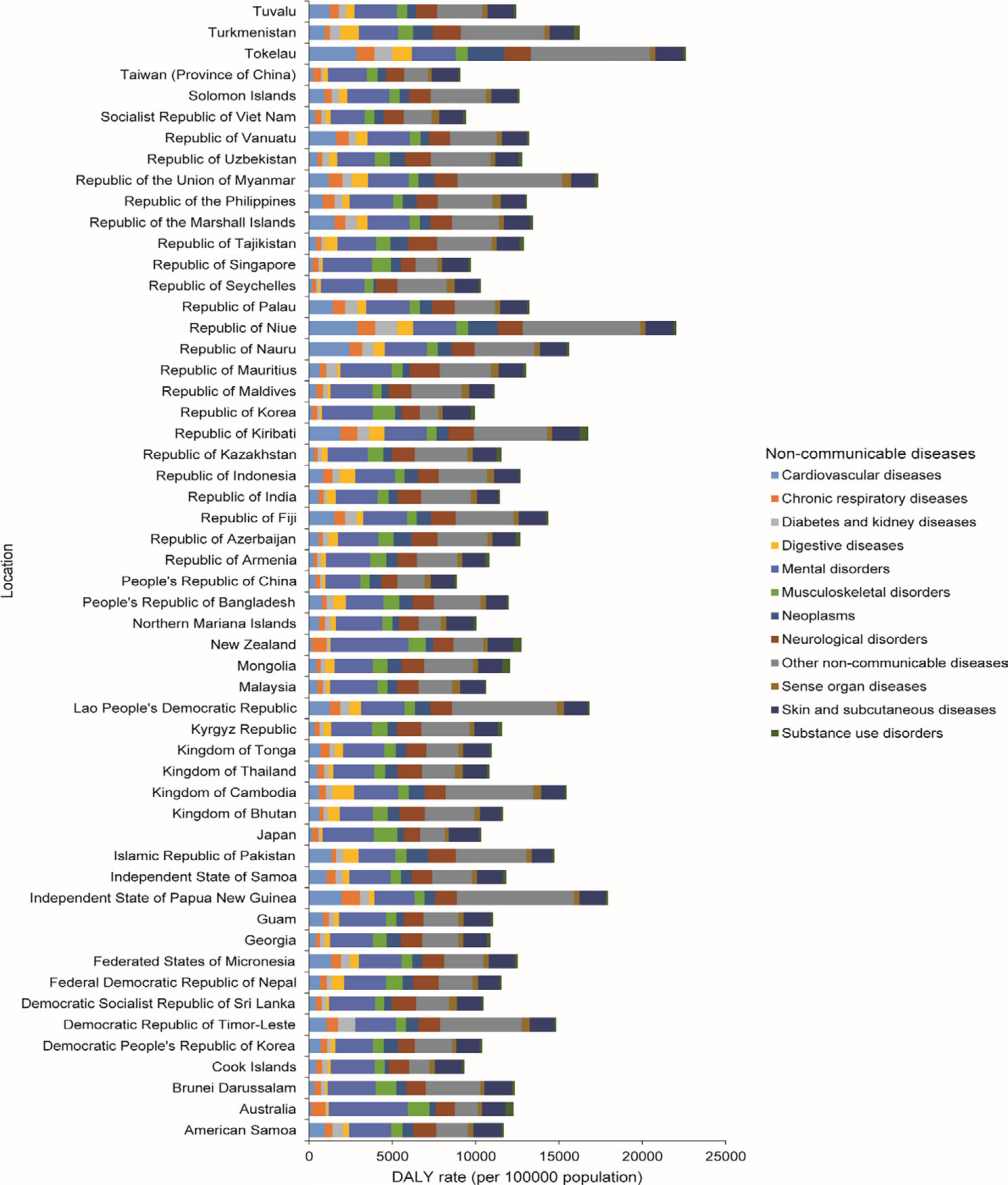
DALY rate per 100,000 population due to level 2 non‑communicable diseases among children and adolescents aged 0–19 years in both sexes, by country in 2021.

In 2021, the highest and lowest DALY rates per 100,000 population due to NCDs in children and adolescents were observed in Tokelau and China, respectively (Figure 4). Other NCDs were the leading level 2 cause of the DALY rate in the Asia‑Pacific region, except in Taiwan (province of China) and the Republic of Korea, which was mental disorders; and China, Northern Mariana Islands, New Zealand, Malaysia, Japan, the Cook Islands, and Australia were neoplasms.

Between 1990 and 2021, the DALY rates due to NCDs among children and adolescents decreased (Figure S18). Digestive disorders and other NCDs were level 2 NCDs that substantially decreased by 53.09% and 40%, respectively (Table S4).

### Socio‑demographic index

The Republic of Korea (SDI = 0.89) and Guinea (SDI = 0.34) had the highest and lowest SDIs in countries across the Asia‑Pacific region, respectively (Figure S19). Countries with lower SDIs reported a higher burden of NCDs, particularly other NCDs, compared to those with higher SDIs (Figure 4, Figures S8 and S19).

## Discussion

This is the first study to estimate the burden of NCDs among children and adolescents aged 0–19 years in the Asia‑Pacific region using GBD 2021 data. The results show that despite a significant decline in death, DALY, and YLL rates due to NCDs between 1990 and 2021, the rate of YLD is still increasing. The predominant cause of death and YLL rates was other NCDs, followed by neoplasms and cardiovascular diseases, whereas congenital birth defects were the level 3 leading cause. Remarkably, the increase in death, DALY, YLL, and YLD rates due to mental disorders is a big concern. Within mental disorders, anxiety and depressive disorders were the leading contributors to YLD and DALY rates, while eating disorders contributed to YLL rates. Studies have documented that sex differences typically broaden with age [14, 15]. This study found that death rates due to NCDs were higher in males than in females, while the DALYs were more observed in females than in males, with the burden of congenital birth defects observed more in those aged 0–14 years and mental disorders in those aged 15–19 years. This study also found that in 2021, among the seven areas of the Asia‑Pacific region, the highest death rates due to NCDs were observed in Oceania and the lowest in high‑income Asia‑Pacific. Significant differences in NCDs across Asia‑Pacific countries were also assessed. Tokelau has the highest death rate, followed by Niue, while the lowest death rates were observed in the Republic of Korea and Japan. Other NCDs were the leading cause of YLL rates across all countries in the Asia‑Pacific region, except in the Republic of Korea, where it was neoplasms. New Zealand and Australia had the highest YLD rates, and Azerbaijan and China the lowest , with mental disorders leading the burden of YLD rates across all countries in the Asia‑Pacific region.

The current research underscores the need to implement comprehensive strategies to tackle the issue of NCDs in children and adolescents in the Asia‑Pacific region, specifically focusing on reducing these disease burdens. These strategies encompass a holistic, multifaceted public health framework [16] such as providing primary and specialty healthcare services, including specialized training in children and adolescent medicine [17], evidence‑based approaches to prevention [8], and school programs on promoting health [18]. Effective interventions should also integrate physical activity, sleep, sedentary behavior, and eating habits [6].

A report has documented that the mental health of children and adolescents aged 0–18 years is one of the most overlooked health challenges worldwide. It was estimated that 10–20% of children and adolescents suffered from poor mental health before COVID‑19 [19]. In East Asia and the Pacific, one in nine girls and one in seven boys aged 10–19 years experienced mental disorders, and suicide was marked as the third leading cause of death among adolescents aged 15–19 years [20]. Research evidence shows that millions of children and adolescents suffer from psychological distress that might not align with the diagnostic standards for mental disorders, yet significantly affects their health, development, and well‑being [21]. In our study, we found that deaths and YLLs due to mental disorders were substantially increased in the Asia‑Pacific region, with eating disorders being the most significant contributor, and anxiety and depressive disorders contributed to the disabilities. In supporting our findings, a nationally representative study conducted in Malaysia showed that the prevalence of depression was 26.9% among children and adolescents aged 3–17 years old, with a higher prevalence in females than males, and the prevalence increased with age [10]. To support and strengthen mental health and psychological support systems and services among children and adolescents in the region, UNICEF in East Asia and the Pacific region and other stakeholders established a regional framework that outlines a tiered multisectoral package of mental health and psychological support systems and services tailored to the specific needs of children and adolescents [21].

Furthermore, our study found that even though the death and disability rates due to other NCDs, particularly congenital birth defects, have substantially decreased, congenital birth defects remain the leading cause of death and disabilities in children and adolescents aged 0–19 years in the Asia‑Pacific region. The burden of congenital birth defects is more pronounced in countries with lower SDIs compared to those with higher SDIs, similar to the previous GBD study [22]. The reason for the lower burden in countries with higher SDIs may be due to easier access to advanced medical technology, which helps improve the survival of children born with congenital disorders [23]. Bridging this gap by enhancing healthcare services and resources for disadvantaged communities must be a key priority in public health [24].

This study also found that apart from other NCDs, neoplasms and cardiovascular diseases were among the top three causes of death for children and adolescents in the Asia‑Pacific region. Similarly, a GBD 2019 study found that neoplasms, cardiovascular diseases, and mental disorders were the leading causes of NCDs among adolescents aged 10–24 years across 42 countries [9]. To reduce the risk of NCDs in children and adolescents, experts in the Asia‑Pacific region have provided a consensus statement on integrated 24‑h activity guidelines for children and adolescents aged 5–18 years [6]. This approach was adopted because children and adolescents in the Asia‑Pacific region were reported to be not adequately physically active compared to other parts of the world [6], with only 6% achieving the WHO’s target of 60 min of MVPA each day [7].

This study has some key limitations. First, the unreliability of data from distinct regions, timespans, and age groups can impact the precision of our estimates, especially due to poor data quality and coverage in the Asia‑Pacific regions. Second, the accuracy of cause‑of‑death and verbal autopsy data depends on the precise coding of death certificates according to international standards established by the International Classification of Diseases. This accuracy is contingent upon the practices of certifying physicians, who may lack the training to ensure consistent reporting of the underlying causes of death. Third, the GBD 2021 study did not account for some sources of uncertainty, such as covariates in the models during the estimation process, which could lead to confounding or selection bias.

## Conclusions

Our study shows that despite a significant decline in deaths and disabilities due to NCDs between 1990 and 2021, the increase in deaths and disabilities attributed to mental disorders raises serious concerns across the Asia‑Pacific region. Additionally, considerable gaps remain evident with locations, sex, age groups, and socioeconomic classes. The insight gained from our study guides resource distribution, public health strategies, and focused interventions to improve the prevention and management of NCDs in the Asia‑Pacific region.

## Data Availability

The datasets analyzed during the current study are available in the Global Health Data Exchange repository (https://vizhub.healthdata.org/gbd-results/). Further data can be requested from the corresponding author.

## Additional File

The additional file for this article can be found as follows:

Supplementary material. Supplementary appendix.

This finding of multiple local introgression events has at least three key implications.
