# Supplementary material for: Burden of Non‑Communicable Diseases Among Children and Adolescents in the Asia‑Pacific Region, 1990–2021: Analysis for the Global Burden of Diseases Study 2021
Source: Ann Glob Health. 2025 Dec 17;91(1):88. doi: 10.5334/aogh.4891 (PMC12716244; doi:10.5334/aogh.4891)
Supplement: Supplementary material. — Supplementary appendix. [file agh-91-1-4891-s1.pdf]

Supplementary appendix

Supplement to: Burden of Non-Communicable Diseases among Children and Adolescents in the Asia-Pacific Region, 1990-2021: A Systematic Analysis for the Global Burden of Diseases Study 2021

Table of contents

Supplemental Method ... 2

Table S1. Global Burden of Disease 2021 Cause Hierarchy ... 4

Table S2. List of countries by region ... 7

Table S3. Death, YLL, YLD, and DALY rates per 1000000 population among people aged 0-19 years in the Asia-Pacific region by age group and sex in 2021 ... 9

Table S4. Death and DALY rates per 100000 population in children and adolescents aged 0-19 years, and percentage change between 1990 and 2021 ... 12

Table S5. YLL and YLD rates per 100000 population in children and adolescents aged 0-19 years, and percentage change between 1990 and 2021 ... 16

Figure S1. All-cause death rate per 100000 population due to NCDs in the Asia-Pacific region from 1990 to 2021 by age group and sex. ... 20

Figure S2. All-cause death rate per 100000 population due to NCDs in the Asia-Pacific region from 1990 to 2021 by age group and sex. A: Both sexes. B: Female. C: Male. ... 21

Figure S3. Death rate per 100000 population due to NCDs in the Asia-Pacific region in 2021 by sex and age group. A: 0-14 years.B: 15-19 years. ... 22

Figure S4. Death rate per 100000 population due to NCDs in the Asia-Pacific region from 1990 to 2021 ... 23

Figure S5. All-cause YLL rate per 100000 population aged 0-19 years from 1990 to 2021 by sex and age group ... 24

Figure S6. YLL rate per 1000000 population due to NCDs in Asia-Pacific region in 2021 by age group and sex. A: 0-14 years. B: 15-19 years. ... 25

Figure S7. YLL rate per 100000 population due to the first 20 level 3 NCDs among people aged 0-19 years old in the Asia-Pacific region in 2021 by sex. ... 26

Figure S8. YLL rate per 100000 population due to NCDs among people aged 0-19 years old in the Asia-Pacific region in 2021 in both sexes by location. ... 27

Figure S9. YLL rate per 100,000 people due to NCDs among people aged 0-19 years in the Asia-Pacific region in both sexes from 1990 to 2021 ... 28

Figure S10. All-cause YLD rate per 100000 population among people aged 0-19 years from 1990-2021 by sex and age group ... 29

Figure S11. YLD rate per 100000 population due to NCDs among people aged 0-19 years in the Asia-Pacific region in 2021 by sex and age group. A: 0-14 years. B: 15-19 years ... 30

Figure S12. YLD rate per 100000 population due to the first 20 level 3 NCDs among people aged 0-19 years old in the Asia-Pacific region in 2021 by sex. 31

Figure S13. YLD rate per 100000 population due to NCDs among people aged 0-19 years old in the Asia-Pacific region in 2021 in both sexes by location. 32

Figure S14. YLD rate per 100,000 people due to NCDs among people aged 0-19 years in the Asia-Pacific region in both sexes from 1990 to 2021. ... 33

Figure S15. All-cause DALY rate per 100000 population among people aged 0-19 years from 1990-2021 by sex and age group. ... 34

Figure S16. DALY rate per 100000 population due to NCDs among people aged 0-19 years old in the Asia-Pacific region in 2021 by sex. ... 35

Figure S17. DALY rate per 100000 population due to the first 20 level 3 NCDs among people aged 0-19 years old in the Asia-Pacific region in 2021 by sex. ... 36

Figure S18. DALY rate per 100000 population due to NCDs among people aged 0-19 years old in the Asia-Pacific region in both sexes from 1990 to 2021.37

Figure S19. Socio-demographic Index (SDI) in 2021 for countries in the Asia-Pacific region. ... 38

Supplemental research checklist: Revealing compliance with the Guidelines for Accurate and Transparent Health Estimates Reporting (GATHER) ... 39

References ... 41

Supplemental Method

Overview

Global Burden of Disease (GBD) performs systematic reviews, and diligent searches, and also uses data shared by country collaborators and the WHO to get data used in the models. Data collection is an iterative and progressive process to determine new sources. The detailed information for each cause of disease burden can be accessed in a searchable online tool. GBD 2021 estimated the disability-adjusted life years (DALYs), years of life (YLLs), years lived with disability (YLDs), incidence, and prevalence, and healthy life expectancy (HALE) for 371 diseases and injuries. The estimates were generated by age (25 age group from birth to 95 years and above), sex (females, males, and both), year (1990-2021), and location (204 countries and territories grouped into 21 regions and seven super-regions). In addition, the estimates were reported by the Socio-demographic Index.[1] This paper follows the GBD protocol. The estimates are reported in terms of DALYs, YLLs, and YLDs. The reporting followed the Guidelines for Accurate and Transparent Health Estimates Reporting Statement (GATHER) [2].

Geographical location

GBD 2021 generated estimates for 204 countries and territories grouped into 21 regions and seven super-regions. GBD regions, as well as super-regions, consist of nations and territories that are spatially proximate, exhibit epidemiological similarities, and experience comparable patterns of mortality causes. This paper is focused on the Asia-Pacific region. Asia-Pacific region comprises four GBD super-regions including Southeast Asia, East Asia, and Oceania; South Asia; Central Europe, Eastern Europe, and Central Asia; and High-Income (Western Europe, Southern Latin America, North America, Asia Pacific including Australasia). In the current study, we produced the estimates for Asia-Pacific regions including seven GBD regions namely Australasia, Central Asia, East Asia, High-income Asia Pacific, Oceania, South Asia, and Southeast Asia (**Figure 1, Table S2**).

Disease and injury

The GBD 2021 causes and injuries list is described in a four-level hierarchy that includes both fatal and non-fatal causes, each level within a category is unique and no item can belong to more than one level. The first-level causes of disease burdens include communicable, maternal, neonatal, and nutritional status (CMNN); non-communicable diseases (NCDs); and injuries. In the current study, we reported all three level 1 causes of disease burdens to provide a comprehensive understanding of the causes; CMNN, NCDs, and injury. We then focused on levels 2 and 3 causes of NCDs, which was the aim of our study. The second-level NCDs highlighted in GBD 2021 are Neoplasms; cardiovascular diseases; chronic respiratory diseases; digestive diseases; neurological disorders; mental disorders; substance use disorders; diabetes and kidney diseases; skin and subcutaneous diseases; sense organ diseases; musculoskeletal disorders; and other non-communicable diseases (congenital birth defects; urinary diseases and male infertility; gynecological diseases; hemoglobinopathies and hemolytic anemias; endocrine, metabolic, blood, and immune disorders; oral disorders; and sudden infant death syndrome). The detailed GBD 2021 Cause Hierarchy is presented in **Table S1**. In this study, the NCDs were identified using disability-adjusted life years (DALY), years of life lost (YLLs), and years of lived with disability (YLDs) data from the GBD 2021. The GBD 2021 uses DALYs to measure the population's disease burden by taking the sum of YLLs due to premature mortality and YLDs, which integrate fatal and non-fatal disease burden. The estimates produced in the GBD 2021 were presented with 95% uncertainty intervals (UIs). The DALYs, YLDs, and YLLs for each NCD were estimated by age (for 25 age groups from birth to 95 years and older), sex (for females, males, and both), year (from 1990-2021), and location (for 204 countries and territories grouped into 21 regions and seven super-regions). The age-standardized were computed according to the GBD population age standard. We presented estimates as age-standardized and all-age results to allow comparison of estimates of different age structures within the population and percentage changes between 1990 and 2021. In addition, the YLDs and YLLs were also estimated.

Data sources

In the GBD 2021, the estimation of DALYs was based on the information from 100983 data sources while that of YLLs was based on the data sources discovered in the previous cycles along with 9248 newly identified sources. Multiple data types were included to capture the large body of information such as vital registration and verbal autopsy, census, survey, surveillance, cancer registry, open-source databases, police records, and minimally invasive tissue sampling. Data were standardized by applying a set of data processing corrections to allow comparison by cause, age, sex, location, and time. The vital registration and verbal autopsy were assessed for completeness. Sources with

less than 50 percent completeness were excluded. The details about GBD 2021 data sources and processing have been described in the previous studies [1, 3].

DALYs, YLLs, and YLDs

DALYs were calculated by the sum of YLLs and YLDs and were estimated by cause, age, sex, location, and year.

YLLs were computed by multiplying the number of deaths for each cause, age, sex, location, year, and the standard life expectancy at each age. The standard life expectancy is computed using the minimum age-specific mortality rate observed across different countries. GBD 2021 used the International Classification of Diseases version 11 (ICD-11) to assign each cause leading to death. Deaths assigned garbage codes were allocated to redistribution algorithms according to the GBD purpose. The GBD 2021 used the Cause of Death Ensemble model (CODEm) to estimate the cause-specific death rates. Alternative approaches were applied to model causes with scant data, major changes in reporting methods for the study, or unusual epidemiology. The detailed methods can be found elsewhere [3].

YLDs were computed as the prevalence for each age, sex, location, and year multiplied by the corresponding disability weights. Network meta-regressions using meta-regression -Bayesian, regulated, trimmed (MR-BRT) were used to estimate correction factors from epidemiologic data with recognizable biases. DisMod-MR 2.1 was used to estimate the prevalence of diseases from 1990 to 2021 and in some cases, spatiotemporal Gaussian process regression was applied instead of DisMod-MR 2.1 [1].

SDI

The SDI is a composite indicator of development made up of the geometric mean of three indicators including income per capita, average years of schooling, and fertility rate among females younger than 25 years for a given location. The SDI scores were scaled from 0 to 1, whereby 0 indicates the lowest income, lowest years of schooling, and the highest fertility and 1 indicates the highest income, highest years of schooling, and lowest fertility. The details process is described elsewhere [1].

Table S1. Global Burden of Disease 2021 Cause Hierarchy

Level 1	Level 2	Level 3
**Communicable, maternal, neonatal, and nutritional status**		
**Non-communicable diseases**		
	**Neoplasms**	
		Lip and oral cavity cancer
		Nasopharynx cancer
		Other pharynx cancer
		Esophageal cancer
		Stomach cancer
		Colon and rectum cancer
		Liver cancer
		Gallbladder and biliary tract cancer
		Pancreatic cancer
		Larynx cancer
		Tracheal, bronchus, and lung cancer
		Malignant skin melanoma
		Non-melanoma skin cancer
		Soft tissue and other extraosseous sarcomas
		Malignant neoplasm of bone and articular cartilage
		Breast cancer
		Cervical cancer
		Uterine cancer
		Prostate cancer
		Ovarian cancer
		Testicular cancer
		Kidney cancer
		Bladder cancer
		Brain and central nervous system cancer
		Eye cancer
		Neuroblastoma and other peripheral nervous cell tumors
		Thyroid cancer
		Mesothelioma
		Hodgkin lymphoma
		Non-Hodgkin lymphoma
		Multiple myeloma
		Leukemia
		Other malignant neoplasms
		Other neoplasms
	**Cardiovascular diseases**	
		Rheumatic heart disease
		Ischemic heart disease
		Stroke
		Hypertensive heart disease
		Non-rheumatic valvular heart disease
		Cardiomyopathy and myocarditis
		Pulmonary Arterial Hypertension
		Atrial fibrillation and flutter
		Aortic aneurysm
		Lower extremity peripheral arterial disease
		Endocarditis
		Other cardiovascular and circulatory diseases
	**Chronic respiratory diseases**	
		Chronic obstructive pulmonary disease
		Pneumoconiosis

		Asthma
		Interstitial lung disease and pulmonary sarcoidosis
		Other chronic respiratory diseases
	**Digestive diseases**	
		Cirrhosis and other chronic liver diseases
		Upper digestive system diseases
		Appendicitis
		Paralytic ileus and intestinal obstruction
		Inguinal, femoral, and abdominal hernia
		Inflammatory bowel disease
		Vascular intestinal disorders
		Gallbladder and biliary diseases
		Pancreatitis
		Other digestive diseases
	**Neurological disorders**	
		Alzheimer's disease and other dementias
		Parkinson's disease
		Idiopathic epilepsy
		Multiple sclerosis
		Motor neuron disease
		Headache disorders
		Other neurological disorders
	**Mental disorders**	
		Schizophrenia
		Depressive disorders
		Bipolar disorder
		Anxiety disorders
		Eating disorders
		Autism spectrum disorders
		Attention-deficit/hyperactivity disorder
		Conduct disorder
		Idiopathic developmental intellectual disability
		Other mental disorders
	**Substance use disorders**	
		Alcohol use disorders
		Drug use disorders
	**Diabetes and kidney diseases**	
		Diabetes mellitus
		Chronic kidney disease
		Acute glomerulonephritis
	**Skin and subcutaneous diseases**	
		Dermatitis
		Psoriasis
		Bacterial skin diseases
		Scabies
		Fungal skin diseases
		Viral skin diseases
		Acne vulgaris
		Alopecia areata
		Pruritus
		Urticaria
		Decubitus ulcer
		Other skin and subcutaneous diseases
	**Sense organ diseases**	
		Blindness and vision loss
		Age-related and other hearing loss

		Other sense organ diseases
	**Musculoskeletal disorders**	
		Rheumatoid arthritis
		Osteoarthritis
		Low back pain
		Neck pain
		Gout
		Other musculoskeletal disorders
	**Other non-communicable diseases**	
		Congenital birth defects
		Urinary diseases and male infertility
		Gynecological diseases
		Hemoglobinopathies and hemolytic anemias
		Endocrine, metabolic, blood, and immune disorders
		Oral disorders
		Sudden infant death syndrome
Injuries		

Table S2. List of countries by region

Region	Country
**Australasia**	
	Australia
	New Zealand
**Central Asia**	
	Armenia
	Azerbaijan
	Georgia
	Kazakhstan
	Kyrgyzstan
	Mongolia
	Tajikistan
	Turkmenistan
	Uzbekistan
**East Asia**	
	China
	Democratic People's Republic of Korea
	Taiwan (Province of China)
**High-income Asia Pacific**	
	Brunei Darussalam
	Japan
	Republic of Korea
	Singapore
**Oceania**	
	Fiji
	Kiribati
	Marshall Islands
	Micronesia (Federated States of)
	Papua New Guinea
	Samoa
	Solomon Islands
	Tonga
	Vanuatu
**South Asia**	
	Bangladesh
	Bhutan
	India
	Nepal
	Pakistan
	Mauritius
	Seychelles
	American Samoa
	Cook Islands
	Guam
	Nauru
	Niue
	Northern Mariana Islands
	Palau
	Tokelau
	Tuvalu
**Southeast Asia**	
	Cambodia

	Indonesia
	Lao People's Democratic Republic
	Malaysia
	Maldives
	Myanmar
	Philippines
	Sri Lanka
	Thailand
	Timor-Leste
	Viet Nam

Table S3. Death, YLL, YLD, and DALY rates per 1000000 population among people aged 0-19 years in the Asia-Pacific region by age group and sex in 2021

		Both	Female	Male
Death				
Age	cause	Rate (95% UI)	Rate (95% UI)	Rate (95%UI)
Death				
**0-6 days**	CMNN	242110.75 (202941.20, 288445.90)	212294.27 (179401.87, 250919.00)	269551.89 (223514.75, 324570.87)
	NCDs	33465.80 (23076.57, 44607.73)	30478.14 (20256.20, 42574.18)	36217.12 (23102.05, 51039.68)
	Injuries	1347.60 (1000.29, 1794.31)	1288.36 (899.24, 1765.88)	1402.10 (987.36, 1939.57)
**7-27 days**	CMNN	21210.24 (17872.54, 25010.87)	21950.91 (18458.33, 25780.24)	20545.11 (16963.69, 24688.37)
	NCDs	4606.34 (3364.55, 5903.51)	4275.96 (2979.08, 5795.79)	4910.56 (3452.69, 6552.42)
	Injuries	367.37 (265.73, 474.12)	380.91 (263.37, 506.07)	355.43 (248.57, 465.66)
**1-5 months**	CMNN	3451.37 (2846.17, 4142.91)	3101.79 (2550.38, 3697.82)	3771.64 (3057.37, 4621.26)
	NCDs	1594.17 (1186.03, 2023.61)	1393.67 (1051.45, 1805.03)	1777.92 (1250.71, 2362.29)
	Injuries	297.96 (221.49, 376.14)	276.11 (201.57, 357.82)	317.86 (219.58, 421.61)
**6-11 months**	CMNN	2294.17 (1751.62, 2952.28)	2215.06 (1674.84, 2860.79)	2366.49 (1764.07, 3098.45)
	NCDs	816.97 (495.87, 1152.26)	792.56 (484.90, 1139.99)	91.85 (65.13, 124.63)
	Injuries	200.26 (139.30, 277.26)	185.47(126.03, 266.97)	213.73(142.14, 302.09)
**<1 year**	CMNN	8529.16(7132.45, 10116.45)	7803.02(6607.15, 9186.06)	9198.72(7592.46, 11091.33)
	NCDs	1996.56(1434.08, 2548.60)	1821.88(1293.13, 2391.07)	2156.98(1484.18, 2888.41)
	Injuries	272.92(205.03, 350.96)	255.889(188.48, 332.42)	288.47(204.94, 377.74)
**2-4 years**	CMNN	191.00(146.56, 245.45)	183.17(139.33, 236.30)	198.28(150.62, 257.84)
	NCDs	72.34(52.14, 96.85)	68.80(48.64, 94.31)	75.64(52.91, 102.85)
	Injuries	80.70(59.09, 106.99)	55.44(40.44, 74.06)	104.01(73.97, 141.93)
**5-9 years**	CMNN	92.80(77.36, 109.70)	90.84(75.44, 108.91)	94.66 (78.35, 113.55)
	NCDs	69.09 (57.84, 81.57)	62.94(51.76, 75.54)	74.75(61.38, 89.66)
	Injuries	74.74(62.77, 87.88)	48.26(39.65, 56.99)	99.03 (82.12, 117.62)
**10-14 years**	CMNN	75.97(63.88, 90.01)	76.89(63.77, 92.15)	75.20(62.00, 90.61)
	NCDs	75.12(65.39, 84.89)	76.25(64.07, 88.03)	74.14(63.95, 85.20)
	Injuries	75.45 (65.95, 85.90)	48.58(41.32, 56.40)	100.04(86.34, 115.74)
**0-14 years**	CMNN	745.11(623.14, 885.92)	692.49(585.59, 814.34)	793.80(654.38, 957.68)
	NCDs	223.22(165.70, 279.28)	209.19(156.21, 264.49)	236.18(169.99, 305.26)
	Injuries	102.02(83.66, 122.94)	73.59(60.32, 89.21)	128.10(104.39, 156.41)
**15-19 years**	CMNN	104.19(85.10, 127.90)	121.61(97.99, 149.51)	88.30(70.61, 111.22)
	NCDs	133.23(114.57, 153.99)	127.10(105.86, 151.56)	138.85(118.24, 163.25)
	Injuries	198.79(176.79, 222.27)	115.42(100.45, 132.10)	276.02(241.09, 313.86)
**0-19 years**	CMNN	849.30(708.24, 1013.82)	814.10 (683.57, 963.85)	882.11(725.00, 1068.91)
	NCDs	356.45(280.27, 433.27)	336.29(262.07, 416.05)	375.03(288.24, 468.51)
	Injuries	300.81(260.44, 345.21)	189.01(160.78, 221.31)	404.12(345.48, 470.27)
YLLs				
		Both	Female	Male
Age	cause	Rate (95% UI)	Rate (95% UI)	Rate (95%UI)
**0-6 days**	CMNN	21784029.16 (25953056.68, 18259730.53)	19101277.41 (16141768.61, 22576556.38)	24253059.17 (20110845.42, 29203418.09)
	NCDs	3011101.57 (4013601.51, 2076325.14)	2742284.75(1822561.62, 3830631.73)	3258652.82(2078617.77, 4592319.14)
	Injuries	121251.34(161443.83, 90001.76)	115920.98(80909.27, 158885.81)	126154.53(88838.41, 174513.40)
**7-27 days**	CMNN	1908354.68(1608050.26, 2250309.12)	1974994.52(1660755.90, 2319532.41)	1848510.13(1526278.07, 2221293.10)
	NCDs	414447.19(302719.48, 531158.21)	384722.28(268037.93, 521466.15)	441819.42(310649.56, 589542.82)
	Injuries	33053.69 (23908.31, 42658.35)	34271.50(23696.29, 45532.58)	31978.79(22364.92, 41896.71)
**1-5 months**	CMNN	309797.11(255474.19, 371869.61)	278418.32(228923.59, 331918.49)	338544.69(274431.07, 414806.90)
	NCDs	143094.00(106458.75, 181640.63)	125096.97(94378.73, 162020.93)	113615.53(91169.29, 142106.46)
	Injuries	26744.69(19881.08, 33762.63)	24783.63(18093.28, 32118.48)	28531.50(19710.01, 37843.57)
**6-11 months**	CMNN	204914.12(156453.63, 263695.86)	197847.64(149596.10, 255524.45)	211373.97(157565.65, 276752.32)
	NCDs	72971.10(44290.88, 102919.07)	70790.79(43310.87, 101823.43)	74940.50(41573.42, 111369.54)
	Injuries	17886.75(12442.42, 24764.51)	16566.22(11256.87, 23845.32)	19090.49(12696.25, 26982.94)

**<1 year**	CMNN	766349.30(640862.90, 908974.00)	701072.22(593668.18, 825302.18)	826540.85(682240.95, 996531.02)
	NCDs	179228.63(128757.60, 228758.64)	163537.90(116093.97, 214613.39)	193638.73(133258.70, 259279.84)
	Injuries	24463.15(18377.86, 31453.63)	22937.38(16896.62, 29794.18)	25856.59(18371.83, 33855.96)
**2-4 years**	CMNN	16540.47(12692.18, 21256.19)	15862.55(12066.27, 20464.21)	17170.97(13044.06, 22329.11)
	NCDs	6264.31(4515.37, 8386.56)	5957.41(4211.60, 8167.11)	6549.97(4581.80, 8906.27)
	Injuries	6988.08(5116.69, 9265.17)	4800.97(3502.16, 6413.19)	9006.56(6405.21, 12290.64)
**5-9 years**	CMNN	7687.11(6407.99, 9087.18)	7527.64(6251.52, 9025.47)	7837.83(6487.35, 9402.21)
	NCDs	5722.20(4790.91, 6756.18)	5215.92(4288.76, 6259.93)	6189.03(5081.66, 7423.79)
	Injuries	6190.22(5198.67, 7279.37)	3999.66(3285.39, 4722.93)	8200.83(6800.40, 9740.36)
**10-14 years**	CMNN	5888.98(4951.31, 6977.36)	5960.97(4944.14, 7144.29)	5828.26(4805.15, 7022.24)
	NCDs	5821.02(5066.77, 6578.23)	5910.58(4966.06, 6823.45)	5743.36(4954.01, 6600.51)
	Injuries	5846.05(5109.82, 6656.07)	3764.77(3202.20, 4371.05)	7750.48(6688.80, 8966.64)
**0-14 years**	CMNN	66213.59(55360.09, 78795.38)	61493.22(51964.73, 72377.27)	70581.81(58154.95, 85214.42)
	NCDs	19501.85(14393.17, 24467.23)	18256.20(13548.42, 23152.61)	20652.16(14774.86, 26773.21)
	Injuries	8605.77(7034.86, 10399.51)	6244.09(5098.98, 7604.07)	10772.54(8746.47, 13201.24)
**15-19 years**	CMNN	7554.73(6170.74, 9274.40)	8819.25(7106.34, 10843.02)	6401.80(5118.95, 8063.84)
	NCDs	9657.38(8305.13, 11163.23)	9217.49(7676.61, 10990.65)	10061.60(8567.92, 11830.75)
	Injuries	14406.75(12811.82, 16109.26)	8369.01(7284.05, 9579.35)	19999.84(17467.90, 22742.53)
**0-19 years**	CMNN	73768.32 (61530.83, 88069.78)	70312.47 59071.07 83220.28	76983.61 (63273.91, 93278.26)
	NCDs	29159.23 (22698.30, 35630.45)	27473.68 (21225.03, 34143.26)	30713.76 (23342.78, 38603.96)
	Injuries	23012.51 (19846.68, 26508.76)	14613.10 (12383.03, 17183.41)	30772.37 (26214.37, 35943.77)
**YLDs**				
		**Both**	**Female**	**Male**
**Age**	**cause**	**Rate (95% UI)**	**Rate (95% UI)**	**Rate (95%UI)**
**0-6 days**	CMNN	12288.42(8300.44, 18149.28)	14332.01(9499.26, 21797.16)	10407.43(6809.25, 16738.42)
	NCDs	8461.79(6300.13, 11092.35)	8041.43(5878.84, 10692.11)	8846.04(6628.75, 11535.30)
	Injuries	474.13(321.60, 664.28)	491.62(333.98, 695.21)	457.22(310.34, 634.86)
**7-27 days**	CMNN	15256.68(9637.96, 23101.43)	14556.43(8787.98, 23187.74)	15915.48(8747.55, 26114.33)
	NCDs	7761.55(5767.40, 10193.48)	7156.97(5223.79, 9588.79)	8319.43(6212.83, 10879.46)
	Injuries	414.25(282.87, 572.54)	399.83(271.55, 556.35)	426.87(290.99, 589.39)
**1-5 months**	CMNN	13720.75(9525.39, 19299.30)	13011.08(8911.31, 18548.91)	14375.76(9647.10, 20876.82)
	NCDs	7623.75(5665.05, 10070.89)	7032.96(5152.14, 9436.58)	8169.42(6113.76, 10692.02)
	Injuries	374.59(255.41, 519.22)	381.12(259.87, 531.03)	367.87(250.21, 508.31)
**6-11 months**	CMNN	13871.99(9660.20, 19493.29)	13028.38(9047.71, 18521.25)	14650.71(10043.57, 20955.74)
	NCDs	7879.96(5876.48, 10493.32)	7286.61(5348.79, 9864.13)	8429.21(6300.08, 11150.91)
	Injuries	335.80(230.23, 466.40)	344.78(235.30, 481.40)	326.93(224.08, 456.44)
**<1 year**	CMNN	13857.59(9706.95, 19256.53)	13135.83(9170.98, 18361.91)	14524.50(10123.44, 20543.56)
	NCDs	7823.98(5850.07, 10339.06)	7186.85(5296.49, 9645.98)	8321.19(6261.01, 10876.25)
	Injuries	311.55(213.22, 432.86)	366.03(251.00, 511.10)	352.36(241.32, 487.40)
**2-4 years**	CMNN	10045.04(7007.91, 14003.56)	10216.99(7050.37, 14291.78)	10994.60(7545.23, 15860.03)
	NCDs	12815.85(9004.61, 17559.54)	12281.56(8491.00, 17045.63)	13311.32(9434.42, 18070.18)
	Injuries	559.55(389.54, 789.97)	563.28(389.34, 800.46)	555.61(387.10, 783.39)
**5-9 years**	CMNN	8396.09(5732.57, 12205.97)	8222.85(5620.39, 12432.34)	8554.88(5659.53, 12818.34)
	NCDs	16212.95(11603.78, 22205.71)	15939.14(11310.82, 22092.98)	16465.79(11868.56, 22319.25)
	Injuries	1056.36(740.02, 1472.00)	1046.02(729.31, 1457.42)	1065.94(744.72, 1485.56)
**10-14 years**	CMNN	7199.40(5089.17, 9879.86)	7735.74(5406.07, 10798.70)	6700.17(4719.03, 9263.12)
	NCDs	26893.60(19251.15, 36701.28)	29000.38(20555.25, 39955.55)	24938.10(17885.72, 33735.27)
	Injuries	1722.41(1231.30, 2361.72)	1672.81(1188.92, 2285.27)	1769.76(1264.24, 2446.41)
**0-14 years**	CMNN	9140.21(6513.45, 12697.24)	9088.85(6439.77, 12611.45)	9185.57(6537.52, 12670.42)
	NCDs	18250.37(13284.48, 24618.80)	18675.96(13467.84, 25470.25)	17853.57(13077.69, 23882.62)
	Injuries	1085.55(774.44, 1485.07)	1067.54(759.27, 1459.60)	1102.48(782.85, 1516.52)
**15-19 years**	CMNN	6905.75(4953.99, 9478.13)	8280.49(5720.92, 11800.86)	5624.38(4065.66, 7615.65)
	NCDs	38238.62(27564.78, 51161.99)	43533.79(31159.33, 58766.41)	33313.83(24195.89, 44443.93)
	Injuries	2609.72(1898.91, 3520.44)	2413.59(1745.04, 3270.27)	2795.86(2007.10, 3794.54)
**0-19 years**	CMNN	16045.96(11467.44, 22175.38)	17369.34(12160.69, 24412.30)	14809.96(10603.18, 20286.07)
	NCDs	56488.99 (40849.26, 75780.79)	62209.75(44627.17, 84236.67)	51167.40 (37273.59, 68326.56)

	Injuries	3695.28 (2673.35, 5005.51)	3481.13(2504.32, 4729.87)	3898.34(2789.95, 5311.06)
**DALYs**				
		**Both**	**Female**	**Male**
**Age**	**cause**	**Rate (95% UI)**	**Rate (95% UI)**	**Rate (95%UI)**
**0-6 days**	CMNN	21796317.58(18271159.68, 25965615.17)	19115609.41(16156393.19, 22589168.00)	24263466.60(20121597.45, 29212457.27)
	NCDs	3019563.36 (2084584.95, 4021554.70)	2750326.18 (1830055.84, 3839376.98)	3267498.86 (2087983.14, 4601550.14)
	Injuries	121725.48(90461.29, 161916.62)	116412.60(81420.31, 159373.75)	126611.75(89297.83, 174970.48)
**7-27 days**	CMNN	1923611.36 (1623577.31, 2264195.25)	1989550.96 (1674789.01, 2333674.17)	1864425.60 (1540907.07, 2236595.00)
	NCDs	422208.74(310649.48, 539002.61)	391879.269(275221.34, 528812.66)	450138.85(319113.46, 598109.26)
	Injuries	33467.94(24284.57, 43070.27)	34671.34(24086.42, 45931.92)	32405.66(22800.58, 42316.43)
**1-5 months**	CMNN	323517.86(270008.27, 385639.96)	291429.40(241466.87, 344766.94)	352920.45(288394.59, 429670.27)
	NCDs	150717.75(114287.04, 189133.90)	132129.93(101233.65, 169370.28)	167757.01(120400.03, 220120.11)
	Injuries	27119.28(20286.51, 34144.08)	25164.75(18478.86, 32505.09)	28899.37(20093.70, 38201.04)
**6-11 months**	CMNN	218786.11(170349.21, 279218.52)	210876.02(162554.49, 268847.69)	226024.68(172575.75, 293731.97)
	NCDs	80851.06(51878.97, 111038.01)	78077.40(50252.20, 109129.57)	83369.71(50211.39, 120098.94)
	Injuries	18222.55(12767.35, 25085.57)	16910.99(11594.90, 24213.57)	19417.42(13038.31, 27311.92)
**<1 year**	CMNN	780206.90(654668.75, 923246.77)	714208.05(607682.33, 838468.89)	841065.35(696901.33, 1011277.88)
	NCDs	187004.91(136840.70, 236595.46)	170724.75(123557.25, 221682.39)	201959.92(141816.09, 267628.76)
	Injuries	24822.40(18760.20, 31829.24)	23303.40(17263.01, 30151.74)	26208.96(18722.53, 34228.97)
**2-4 years**	CMNN	27162.92(21785.37, 33682.30)	26079.54(20851.40, 32365.84)	28165.57(22330.63, 35270.47)
	NCDs	19080.16(14684.78, 24496.54)	18238.97(13873.91, 23684.26)	19861.29(15315.40, 25368.41)
	Injuries	7547.63(5626.44, 9917.33)	5364.25(4004.42, 7081.17)	9562.16(6925.92, 12906.32)
**5-9 years**	CMNN	16083.20(13008.04, 20150.62)	15750.49(12576.54, 20016.12)	16392.71(13108.33, 20944.86)
	NCDs	21935.16(17186.00, 28046.74)	21155.06(16329.19, 27493.31)	22654.82(17856.64, 28690.97)
	Injuries	7246.57(6107.31, 8546.35)	5045.689(4179.42, 6002.84)	9266.78(7736.84, 10979.85)
**10-14 years**	CMNN	13088.38(10666.16, 16035.62)	13696.71(17072.73, 10986.56)	12528.43(10147.89, 15364.67)
	NCDs	32714.62(25080.67, 42476.70)	34910.96(46014.76, 26459.05)	30681.46(23664.81, 39497.37)
	Injuries	7568.46(6562.63, 8757.05)	5437.589(6397.88,4604.65)	9520.25(8201.73, 11073.79)
**0-14 years**	CMNN	75353.81(63996.26, 88268.88)	70582.07(60414.39, 82292.30)	79767.399(67188.29, 94749.86)
	NCDs	37752.22(29864.44, 46373.78)	36932.15(29126.23, 45910.86)	38505.73(29802.46, 47533.99)
	Injuries	9691.32(8021.59., 11663.59)	7311.63(6048.58, 8845.80)	11875.02(9783.71, 14434.63)
**15-19 years**	CMNN	14460.48(11767.81, 17706.05)	17099.74(13599.29, 21334.38)	12026.19(9744.40, 14817.30)
	NCDs	47896.00(37085.48, 61097.16)	52751.28(40188.12, 68173.40)	43375.43(34058.91, 54771.76)
	Injuries	17016.47(15118.90, 19180.32)	10782.60(9378.53, 12514.48)	22795.70(19953.97, 26012.57)
**<0-19 years**	CMNN	89814.29(75764.07, 105974.93)	87681.81(74013.68, 103626.68)	91793.57(76932.69, 109567.16)
	NCDs	85648.22(66949.92, 107470.94)	89683.4354(69314.35, 114084.27)	81881.16(63861.37, 102305.76)
	Injuries	26707.792(23140.49, 30843.91)	18094.23(15427.11, 21360.28)	34670.72(29737.68, 40447.20)

Table S4. Death and DALY rates per 100000 population in children and adolescents aged 0-19 years, and percentage change between 1990 and 2021

	Death rate per 100000 population			DALY rate per 100000 population		
**Cause**	**1990**	**2021**	**% Change, 1990-2021**	**1990**	**2021**	**% Change, 1990-2021**
**Non-communicable diseases**	**643.71 (508.48, 758.86)**	**356.45 (280.27, 433.27)**	**-44.63 (-44.88, -42.91)**	**109276.88 (86885.95, 133191.17)**	**85648.22 (66949.92, 107470.94)**	**-21.62 (-22.95, -19.31)**
**Neoplasms**	**91.02 (75.71,106.41)**	**56.88 (47.40, 67.50)**	**-37.51 (-37.40, -36.57)**	**7199.93 (5970.23, 8444.38)**	**4466.17 (3717.52, 5314.43)**	**-37.97 (-37.73, -37.07)**
Lip and oral cavity cancer	0.44 (0.37, 0.51,)	0.41(0.31,0.52)	-5.61 (-15.89,2.48)	32.09 (27.06, 37.60)	30.39 (22.85, 38.66)	-5.31 (-15.57, 2.81)
Nasopharynx cancer	1.26 (1.02, 1.51)	0.64 (0.50, 0.81)	-49.39 (-50.66, -46.28)	93.92 (76.51, 112.93)	47.77 (37.90, 60.97)	-49.13(-50.46, -46.01)
Other pharynx cancer				0.00 (0.00, 0.00)	0.00 (0.00, 0.00)	0.00 (0.00, 0.00)
Esophageal cancer				0.00 (0.00, 0.00)	0.00 (0.00, 0.00)	0.00 (0.00, 0.00)
Stomach cancer	1.64 (1.26, 1.98)	0.75 (0.58, 0.95)	-54.22 (-54.15, -51.68)	119.51 (92.15, 144.05)	54.80 (42.30, 69.69)	-54.15 (-54.10, -51.62)
Colon and rectum cancer	1.62 (1.30, 1.90)	1.00 (0.81, 1.22)	-38.51(-37.81, -35.93)	118.99 (95.44, 139.42)	73.78 (59.82, 90.16)	-37.99 (-37.32, -35.33)
Liver cancer	2.95 (2.24, 3.92)	1.48 (1.12, 2.04)	-49.75 (-49.91, -48.02)	239.33 (181.61, 316.39)	116.85 (88.23, 160.18)	-51.18 (-51.42, -49.37)
Gallbladder and biliary tract cancer	0.77 (0.33, 1.13)	0.38 (0.23, 0.56)	-51.30 (-29.64, -50.64)	0.00 (0.00, 0.00)	0.00 (0.00, 0.00)	0.00 (0.00, 0.00)
Pancreatic cancer	0.17 (0.14, 0.20)	0.15 (0.13, 0.18)	-11.28 (-11.57, -8.65)	12.34 (10.35, 14.60)	10.95 (9.16, 13.34)	-11.28 (-11.56, -8.62)
Larynx cancer				0.00 (0.00, 0.00)	0.00 (0.00, 0.00)	0.00 (0.00, 0.00)
Tracheal, bronchus, and lung cancer	1.54 (1.26, 1.93)	1.10 (0.85, 1.45)	-28.77 (-32.70, -24.88)	112.26 (91.49, 140.21)	79.99 (61.63, 105.40)	-28.75 (-32.64, -24.83)
Malignant skin melanoma	0.38 (0.31, 0.46)	0.18 (0.13, 0.23)	-53.90 (-56.22, -50.91)	29.46 (23.76, 35.29)	14.10 (10.62, 18.13)	-52.15 (-55.30, -48.63)
Non-melanoma skin cancer				0.00 (0.00, 0.00)	0.00 (0.00, 0.00)	0.00 (0.00, 0.00)
Soft tissue and other extraosseous sarcomas	2.15 (1.61, 2.84)	1.39 (1.05, 1.90)	-35.37 (-34.99, -32.88)	171.30 (128.12, 226.43)	108.73 (81.86, 149.46)	-36.53 (-36.10, -33.99)
Malignant neoplasm of bone and articular cartilage	5.59 (4.11, 7.47)	4.97 (3.50, 6.87)	-11.15 (-14.74, -7.99)	421.85 (309.64, 563.35)	375.03 (264.79, 517.59)	-11.10 (-14.48, -8.12)
Breast cancer	0.54 (0.39, 0.75)	0.66 (0.45, 0.98)	22.89 (16.60, 31.47)	40.10 (28.69, 55.64)	49.69 (33.85, 73.55)	23.91 (17.99, 32.18)
Cervical cancer	0.75 (0.56, 0.98)	0.42 (0.31, 0.60)	-44.32 (-44.85, -38.99)	55.88 (41.39, 73.16)	31.43 (23.08, 45.04)	-43.74 (-44.24, -38.44)
Uterine cancer				0.00 (0.00, 0.00)	0.00 (0.00, 0.00)	0.00 (0.00, 0.00)
Prostate cancer				0.00 (0.00, 0.00)	0.00 (0.00, 0.00)	0.00 (0.00, 0.00)!
Ovarian cancer	0.63 (0.46, 0.84)	0.61 (0.44, 0.78)	-2.81 (-2.64, -7.23)	47.67 (34.37, 63.59)	46.85 (33.99, 59.91)	-1.72 (-1.11, -5.78)
Testicular cancer	0.41 (0.33, 0.51)	0.30 (0.23, 0.38)	-27.17 (-27.94, -26.46)	31.57 (25.05, 39.73)	24.01 (18.55, 30.82)	-23.93 (-25.93, -22.43)
Kidney cancer	1.43 (1.13, 1.78)	0.88 (0.69, 1.10)	-38.42 (-38.79, -38.12)	121.52 (95.66, 152.25)	73.94 (57.91, 93.13)	-39.16 (-39.46, -38.83)
Bladder cancer	0.10 (0.07, 0.11)	0.05 (0.04, 0.06)	-43.88 (-40.14, -41.20)	7.17 (5.48, 8.29)	4.10 (3.33, 4.98)	-42.81 (-39.24, -39.94)
Brain and central nervous system cancer	12.35 (9.17, 15.54)	10.26 (8.07, 12.75)	-16.94 (-12.00, -17.95)	991.25 (734.42, 1251.03)	814.98 (640.60, 1014.30)	-17.78 (-12.78, -18.92)
Eye cancer	0.58 (0.29, 0.97)	0.36 (0.18, 0.65)	-37.99 (-36.69, -32.58)	51.42 (25.32, 85.23)	32.44 (16.27, 58.16)	-36.91 (-35.76, -31.76)
Neuroblastoma and other peripheral nervous cell tumors	0.73 (0.59, 0.91)	0.74 (0.57, 0.95)	0.79 (-3.25, 3.97)	62.50 (50.35, 77.86)	62.36 (48.08, 80.29)	-0.22 (-4.51, 3.12)
Thyroid cancer	0.25 (0.20, 0.31)	0.19 (0.14, 0.26)	-22.83 (-27.85, -16.62)	19.78 (15.68, 24.99)	15.80 (11.67, 21.57)	-20.13 (-25.58, -13.66)
Mesothelioma				0.00 (0.00, 0.00)	0.00 (0.00, 0.00)	0.00 (0.00, 0.00)
Hodgkin lymphoma	1.85 (1.21, 2.50)	0.91 (0.64, 1.32)	-50.87 (-46.93, -47.07)	141.51 (92.33, 191.05)	70.08 (49.51, 101.69)	-50.48 (-46.38, -46.77)
Non-Hodgkin lymphoma	7.12 (5.64, 8.73)	4.31 (3.44, 5.50)	-39.39 (-38.99, -36.98)	562.40 (443.88, 690.56)	339.07 (269.88, 433.26)	-39.71 (-39.20, -37.26)
Multiple myeloma				0.00 (0.00, 0.00)	0.00 (0.00, 0.00)	0.00 (0.00, 0.00)
Leukemia	37.26 (28.18, 46.72)	19.62 (14.63, 25.13)	-47.35 (-48.10, -46.21)	2956.21 (2228.86, 3724.29)	1539.51(1145.56, 1977.00)	-47.92 (-48.60, -46.92)
Other malignant neoplasms	9.04 (6.05, 11.85)	5.24 (3.98, 6.80)	-42.00 (-34.21, -42.64)	728.83 (482.66, 959.89)	418.68 (316.97, 544.48)	-42.55 (-34.33, -43.28)
Other neoplasms	0.26 (0.17, 0.42)	0.27 (0.19, 0.42)	6.70 (12.88, -0.34)	31.05 (21.35, 46.74)	30.83 (21.88, 45.42)	-0.70 (2.46, -2.82)
**Cardiovascular diseases**	**81.90 (66.86, 99.09)**	**48.60 (39.68, 59.06)**	**-40.65 (-40.65, -40.40)**	**7183.67 (5959.42, 8607.52)**	**4513.70 (3730.29, 5441.52)**	**-37.17 (-37.41, -36.78)**
Rheumatic heart disease	25.08 (17.36, 34.02)	14.15 (10.10, 19.86)	-43.58 (-41.83, -41.64)	2209.70 (1585.16, 2963.73	1374.80 (999.50, 1904.84)	-37.78 (-36.95, -35.73)
Ischemic heart disease	9.82 (8.11, 11.84)	7.52 (6.16, 9.13)	-23.42 (-24.03, -22.93)	716.88 (592.98, 864.25)	548.37 (449.12, 665.22)	-23.51 (-24.26, -23.03)
Stroke	27.08 (20.79, 35.86)	13.26 (10.06, 17.08)	-51.05 (-51.59, -52.38)	2508.18 (1977.35, 3247.86)	1330.22 (1043.17, 1663.91)	-46.96 (-47.24, -48.77)
Hypertensive heart disease	0.76 (0.43, 1.12)	0.46 (0.29, 0.66)	-40.06 (-33.08, -40.89)	56.49 (32.87, 82.48	34.48 (22.58, 49.53)	-38.96 (-31.32, -39.94)

Non-rheumatic valvular heart disease	0.63 (0.42, 0.97)	0.57 (0.39, 0.82)	-9.82 (-8.06, -15.46)	45.65 (30.78, 70.58)	41.24 (28.38, 59.75)	-9.64 (-7.79, -15.34)
Cardiomyopathy and myocarditis	10.06 (7.49, 13.91)	6.66 (4.73, 9.19)	-33.83 (-36.80, -33.95)	884.00 (660.84, 1215.14)	606.08 (435.19, 829.21)	-31.44 (-34.15, -31.76)
Pulmonary Arterial Hypertension	1.50 (1.00, 2.20)	0.89 (0.61, 1.32)	-41.00 (-39.04, -40.13)	125.82 (83.54, 184.92)	73.04 (50.34, 108.80)	-41.95 (-39.75, -41.16)
Atrial fibrillation and flutter				0.00 (0.00, 0.00)	0.00 (0.00, 0.00)	0.00 (0.00, 0.00)
Aortic aneurysm	0.20 (0.15, 0.27)	0.17 (0.14, 0.23)	-11.49 (-7.31, -15.00)	14.28 (10.86, 19.28)	12.63 (10.07, 16.39)	-11.50 (-7.31, -15.01)
Lower extremity peripheral arterial disease				0.00 (0.00, 0.00)	0.00 (0.00, 0.00)	0.00 (0.00, 0.00)
Endocarditis	2.05 (1.26, 3.00)	1.68 (1.11, 2.56)	-17.85 (-12.02, -14.67)	164.96 (101.08, 241.33)	132.89 (87.89, 201.36)	-19.44 (-13.04, -16.56)
Other cardiovascular and circulatory diseases	4.73 (3.10, 7.29)	3.26 (2.29, 4.90)	-31.04 (-26.00, -32.76)	457.72 (305.62, 695.43)	359.95 (249.17, 541.81)	-21.36 (-18.47, -22.09)
**Chronic respiratory diseases**	**30.95 (21.10, 39.72)**	**16.87 (12.09, 23.61)**	**-45.51 (-42.72, -40.57)**	**5751.21 (4025.25, 7911.99)**	**3653.82 (2477.96, 5323.57)**	**-36.47 (-38.44, -32.72)**
Chronic obstructive pulmonary disease	4.36 (2.99, 5.93)	2.63 (1.79, 3.79)	-39.52 (-40.08, -36.06)	458.18 (342.39, 586.06)	323.18 (242.31, 423.28)	-29.47 (-29.23, -27.78)
Pneumoconiosis	0.02 (0.01, 0.02)	0.01 (0.00, 0.01)	-66.23 (-67.11, -63.72)	1.37 (0.95, 1.93)	0.60 (0.39, 0.91)	-56.12 (-58.74, -52.61)
Asthma	16.80 (11.44, 22.03)	7.42 (5.34, 10.49)	-55.83 (-53.29, -52.40)	4435.53 (3004.61, 6358.78)	2719.25 (1721.22, 4155.42)	-38.69 (-42.71, -34.65)
Interstitial lung disease and pulmonary sarcoidosis	1.23 (0.67, 2.11)	1.25 (0.72, 2.10)	1.44 (7.40, -0.84)	94.66 (51.13, 163.79)	95.25 (54.64, 160.75)	0.62 (6.86, -1.86)
Other chronic respiratory diseases	8.55 (5.17, 12.30)	5.56 (3.27, 8.92)	-35.00 (-36.71, -27.51)	761.47 (467.58, 1080.85)	515.54 (315.76, 803.29)	-32.30 (-32.47, -25.68)
**Digestive diseases**	**59.59 (47.23, 73.68)**	**24.22 (19.26, 29.88)**	**-59.35 (-59.23, -59.45)**	**5630.30 (4553.40, 6861.43)**	**2640.92 (2116.90, 3246.46)**	**-53.09 (-53.51, -52.69)**
Cirrhosis and other chronic liver diseases	23.37 (18.29, 30.36)	11.25 (8.98, 13.91)	-51.88 (-50.89, -54.16)	1862.85 (1464.71, 2407.12)	893.39 (720.59, 1098.98)	-52.04 (-50.80, -54.34)
Upper digestive system diseases	5.32 (3.87, 7.16)	1.70 (1.24, 2.31)	-68.00 (-67.80, -67.70)	704.91 (517.52, 953.72)	412.95 (277.07, 603.22)	-41.42 (-46.46, -36.75)
Appendicitis	4.94 (3.27, 6.75)	1.45 (1.01, 1.96)	-70.72 (-69.24, -70.97)	421.09 (283.67, 575.04)	157.79 (106.72, 221.58)	-62.53 (-62.38, -61.47)
Paralytic ileus and intestinal obstruction	15.83 (11.76, 20.38)	6.43 (4.66, 8.53)	-59.40 (-60.36, -58.14)	1360.45 (1017.33, 1744.79)	549.76 (401.82, 725.59)	-59.59 (-60.50, -58.41
Inguinal, femoral, and abdominal hernia	3.25 (1.73, 5.39)	0.76 (0.47, 1.17)	-76.64 (-73.05, -78.24)	439.25 (267.06, 684.70)	185.56 (119.17, 280.68)	-57.76 (-55.38, -59.01)
Inflammatory bowel disease	0.66 (0.40, 0.96)	0.28 (0.20, 0.37)	-58.02 (-50.01, -61.31)	64.58 (40.09, 94.39)	32.84 (23.32, 44.70)	-49.14 (-41.83, -52.65)
Vascular intestinal disorders	0.21 (0.15, 0.28)	0.11 (0.08, 0.14)	-47.87 (-44.52, -48.72)	18.50 (13.55, 24.46)	10.62 (8.08, 14.05)	-42.60 (-40.38, -42.57)
Gallbladder and biliary diseases	0.77 (0.33, 1.13)	0.38 (0.23, 0.56)	-51.30 (-29.64, -50.64)	277.22 (173.44, 410.24)	210.77 (129.61, 323.37)	-23.97 (-25.27, -21.18)
Pancreatitis	1.14 (0.77, 1.73)	0.71 (0.50, 1.03)	-37.18 (-35.02, -40.39)	98.57 (68.38, 148.24)	65.88 (46.08, 96.01)	-33.17 (-32.61, -35.23)
Other digestive diseases	4.10 (2.62, 5.73)	1.16 (0.82, 1.69)	-71.62 (-68.77, -70.45)	382.89 (256.57, 521.31)	121.35 (90.15,168.26)	-68.31 (-64.86, -67.72)
**Neurological disorders**	**22.56 (17.65, 26.83)**	**17.59 (14.21, 21.68)**	**-22.03 (-19.51, -19.21)**	**8991.31 (3884.46, 17065.74)**	**8643.85 (3434.39, 6830.79)**	**-3.86 (-11.59, -1.38)**
Alzheimer's disease and other dementias				0.00 (0.00, 0.00)	0.00 (0.00, 0.00)	0.00 (0.00, 0.00)
Parkinson's disease				0.00 (0.00, 0.00)	0.00 (0.00, 0.00)	0.00 (0.00, 0.00)
Idiopathic epilepsy	18.28 (13.64, 22.37)	13.26 (10.22, 16.92)	-27.45 (-25.09, -24.36)	2832.13 (1919.97, 4005.39)	2279.94 (1525.60, 3416.59)	-19.50 (-20.54, -14.70)
Multiple sclerosis	0.03 (0.02, 0.04)	0.02 (0.02, 0.03)	-22.66 (-24.00, -22.42)	5.81 (3.90, 8.37)	5.70 (3.63, 8.54)	-1.96 (-6.80, 2.12)
Motor neuron disease	0.63 (0.48, 0.76)	0.38 (0.27, 0.49)	-40.81 (-44.52, -35.99)	60.29 (46.88, 72.30)	37.39 (27.66, 48.12)	-37.97 (-41.00, -33.44)
Headache disorders				5578.64 (595.85, 13346.20)	5640.63(595.86, 13474.86)	1.11 (0.00, 0.96)
Other neurological disorders	3.61(3.11, 4.28)	3.93(3.18, 4.75)	8.71(2.29, 10.96)	514.44 (382.70, 700.09)	680.19 (468.98, 1008.05)	32.22 (22.54, 43.99)
**Mental disorders**	**0.04 (0.03, 0.06)**	**0.05 (0.04, 0.08)**	**29.26 (24.45, 35.97)**	**17753.12 (12828.65, 23513.59)**	**19541.55 (14037.37, 26089.18)**	**10.07 (9.42, 10.95)**
Schizophrenia				380.32 (216.28, 606.86)	390.57 (224.10, 624.37)	2.69 (3.62, 2.89)
Depressive disorders				4020.52 (2507.50, 6040.02)	4714.38 (2856.48, 7226.49)	17.26 (13.92, 19.64)
Bipolar disorder				857.34 (491.45, 1399.29)	859.97 (492.43, 1392.71)	0.31 (0.20, -0.47)
Anxiety disorders				4965.16 (3032.82, 7499.90)	5852.03 (3509.35, 8967.74)	17.86 (15.71, 19.57)
Eating disorders	0.04 (0.03, 0.06)	0.05 (0.04, 0.08)	29.26 (24.45, 35.97)	789.14 (439.56, 1336.38)	995.30 (571.56, 1672.28)	26.12 (30.03, 25.13)
Autism spectrum disorders				2521.90 (1724.61, 3537.78)	2595.71 (1773.60, 3649.42)	2.93 (2.84, 3.16)

Attention-deficit/hyperactivity disorder				429.14 (221.53, 728.87)	449.70 (234.35, 747.88)	4.79 (5.79, 2.61)
Conduct disorder				2718.90 (1447.69, 4312.38)	2801.20 (1478.96,4479.04)	3.03 (2.16, 3.86)
Idiopathic developmental intellectual disability				809.67 (359.08, 1416.30)	620.75 (274.29, 1088.31)	-23.33 (-23.61, -23.16)
Other mental disorders				261.02 (143.70, 419.35)	261.95 (144.24, 420.26)	0.35 (0.38, 0.22)
**Substance use disorders**	**7.26 (6.07, 8.48)**	**3.87 (3.03, 4.86)**	**-46.71 (-50.13, -42.66)**	**1849.03 (1380.27, 2439.70)**	**1399.12 (1004.93, 1913.68)**	**-24.33 (-27.19, -21.56)**
Alcohol use disorders	2.67 (2.15, 3.19)	1.06 (0.68, 1.49)	-60.22 (-68.50, -53.50)	773.86 (519.60, 1135.32)	565.96 (344.44, 875.80)	-26.87 (-33.71, -22.86)
Drug use disorders	4.29 (3.54, 5.17)	2.64 (2.03, 3.39)	-38.57 (-42.81, -34.36)	1075.17 (786.19, 1428.93)	833.16 (582.49, 1139.75)	-22.51 (-25.91, -20.24)
**Diabetes and kidney diseases**	**21.84 (16.38, 26.50)**	**17.06 (13.53, 20.62)**	**-21.86 (-17.38, -22.20)**	**2087.90 (1601.80, 2524.51)**	**1825.37 (1446.79, 2237.24)**	**-12.57 (-9.68, -11.38)**
Diabetes mellitus	5.85 (4.46, 7.42)	4.99 (3.90, 6.28)	-14.76 (-12.53, -15.33)	644.03 (489.84, 831.89)	727.34 (543.06, 971.76)	12.94 (10.86, 16.81)
Chronic kidney disease	14.72 (10.39, 18.16)	11.76 (8.99, 14.58)	-20.09 (-13.47, -19.74)	1342.11 (964.97, 1652.35)	1072.97 (827.36, 1327.71)	-20.05 (-14.26, -19.65)
Acute glomerulonephritis	1.27 (0.80, 1.81)	0.31 (0.18, 0.45)	-75.22 (-76.99, -75.11)	101.76 (64.06, 145.69)	25.06 (14.71, 35.90)	-75.37 (-77.04, -75.36)
**Skin and subcutaneous diseases**	**3.67 (2.61, 5.07)**	**2.03 (1.40, 2.96)**	**-44.78 (-46.31, -41.65)**	**10011.05 (6364.04, 14841.79)**	**10322.86 (6531.77, 15402.83)**	**3.11 (2.64, 3.78)**
Dermatitis				2717.40 (1434.14, 4548.02)	2726.08 (1431.42, 4570.51)	0.32 (-0.19, 0.49)
Psoriasis				255.34 (175.89, 353.84)	294.23 (201.85, 407.77)	15.23 (14.76, 15.24)
Bacterial skin diseases	2.89 (1.90, 4.08)	1.61 (0.94, 2.46)	-44.43 (-50.25, -39.62)	281.62 (188.86, 395.93)	171.41 (106.67, 260.55)	-39.13 (-43.52, -34.19)
Scabies				1386.16 (711.31, 2333.45)	1388.29 (708.76, 2349.49)	0.15 (-0.36, 0.69)
Fungal skin diseases				395.69 (152.80, 847.12)	382.81 (147.99, 817.94)	-3.25 (-3.14, -3.44)
Viral skin diseases				1324.87 (831.25, 2026.91)	1402.01 (876.38, 2149.34)	5.82 (5.43, 6.04)
Acne vulgaris				2296.06 (1403.86, 3695.83)	2584.55(1568.81, 4158.27)	12.56 (11.75, 12.51)
Alopecia areata				60.31 (37.52, 89.79)	60.62 (37.73, 90.39)	0.53 (0.56, 0.67)
Pruritus				78.21 (34.98, 156.38)	85.14 (38.03, 171.36)	8.86 (8.72, 9.57)
Urticaria				898.75 (546.31, 1384.72)	896.34 (542.81, 1383.72)	-0.27 (-0.64, -0.07)
Decubitus ulcer	0.24 (0.11, 0.46)	0.18 (0.07, 0.38)	-24.06 (-39.28, -18.84)	20.39 (10.27, 38.04)	16.12 (7.07, 31.63)	-20.93 (-31.15, -16.85)
Other skin and subcutaneous diseases	0.54 (0.26, 1.04)	0.24 (0.15, 0.46)	-55.74 (-44.98, -55.48)	296.25 (153.48, 519.96)	315.27 (155.92, 565.36)	6.42 (1.59, 8.73)
**Sense organ diseases**				**2266.91 (1500.89, 3257.17)**	**2317.71 (1534.71, 3332.76)**	**2.24 (2.25, 2.32)**
Blindness and vision loss				777.27 (476.67, 1221.13)	748.43 (456.02, 1189.57)	-3.71 (-4.33, -2.58)
Age-related and other hearing loss				1312.79 (823.91, 1969.09)	1386.40 (871.36, 2064.84)	5.61 (5.76, 4.86)
Other sense organ diseases				176.85 (97.36, 291.43)	182.87 (100.20, 301.40)	3.40 (2.92, 3.42)
**Musculoskeletal disorders**	**2.84 (2.01, 3.81)**	**2.20 (1.42, 3.08)**	**-22.70 (-29.40, -19.04)**	**6150.11 (4031.60, 8772.70)**	**5995.95 (3907.95, 8544.82)**	**-2.51 (-3.07, -2.60)**
Rheumatoid arthritis	0.09 (0.07, 0.13)	0.04 (0.03, 0.06)	-54.30 (-56.86, -55.75)	32.21 (19.83, 49.96)	35.70 (20.62, 57.30)	10.82 (4.01, 14.69)
Osteoarthritis				0.00 (0.00, 0.00)	0.00 (0.00, 0.00)	0.00 (0.00, 0.00)
Low back pain				4214.42 (2588.43, 6254.46)	3940.74 (2401.55, 5840.50)	-6.49 (-7.22, -6.62)
Neck pain				826.48 (382.65, 1546.41)	833.03 (384.49, 1560.40)	0.79 (0.48, 0.91)
Gout				0.91 (0.27, 2.04)	1.02 (0.31, 2.28)	11.29 (14.97, 11.80)
Other musculoskeletal disorders	2.75 (1.93, 3.70)	2.15 (1.38, 3.04)	-21.64 (-28.37, -18.00)	1076.09 (668.42, 1668.71)	1185.47 (720.35, 1853.66)	10.16 (7.77, 11.08)
**Other non-communicable diseases**	**322.04 (221.53, 398.90)**	**167.08 (116.63, 216.90)**	**-48.12 -(47.35, -45.62)**	**34402.34 (24610.67, 42625.95)**	**20327.20 (14815.25, 26238.15)**	**-40.91 (-39.80, -38.45)**
Congenital birth defects	272.15 (176.81, 345.78)	145.77 (98.49, 192.40)	-46.44 (-44.29, -44.36)	26009.73 (17404.66, 32709.47)	14789.39 (10355.76, 19134.76)	-43.14 (-40.50, -41.50)
Urinary tract infections and interstitial nephritis	4.03 (2.97, 5.08)	3.05 (2.32, 3.93)	-24.32 (-21.99, -22.74)	350.26 (258.70, 443.38)	266.95 (203.07, 345.30)	-23.79 (-21.50, -22.12)
Gynecological diseases	0.23 (0.13, 0.40)	0.24 (0.13, 0.39)	2.77 (1.84, -3.94)	1475.67 (870.81, 2434.67)	1478.88 (877.18, 2452.72)	0.22 (0.73, 0.74)
Hemoglobinopathies and hemolytic anemias	8.72 (6.14, 11.33)	4.42 (3.04, 5.92)	-49.28 (-50.51, -47.77)	1861.20 (1370.50, 2479.25)	1212.41 (872.97, 1666.40)	-34.86 (-36.30, -32.79)
Endocrine, metabolic, blood, and immune disorders	8.14 (5.75, 10.78)	6.16 (4.59, 8.14)	-24.33 (-20.19, -24.48)	1159.11 (800.69, 1668.02)	955.09 (674.94, 1398.49)	-17.60 (-15.70, -16.16)

Oral disorders				953.15 (517.93, 1591.77)	942.79 (515.17, 1572.50)	-1.09 (-0.53, -1.21)
Sudden infant death syndrome	28.21 (18.26, 41.99)	7.16 (3.81, 11.31)	-74.62 (-79.14, -73.07)	2531.60 (1638.08, 3767.77)	642.54 (341.83, 1014.50)	-74.62 (-79.13, -73.07

Note: Data in parentheses are 95% uncertainty intervals. DALYs, disability-adjusted life year.

Table S5. YLL and YLD rates per 100000 population in children and adolescents aged 0-19 years, and percentage change between 1990 and 2021

Cause	YLL			YLD		
	1990	**2021**	%Change	1990	2021	%Change
**Non-communicable diseases**	**53488.66 (41958.17, 63234.53)**	**29159.23 (22698.30, 35630.45)**	**-45.49 (-45.90, -43.65)**	**55788.22 (40491.03, 75070.89)**	**56488.99 (40849.26, 75780.79)**	**1.26 (0.88, 0.95)**
**Neoplasms**	**7114.92 (5894.84, 8350.54)**	**4378.01 (3643.71, 5208.37)**	**-38.47 (-38.19, -37.63)**	**85.01 (58.20, 117.77)**	**88.16 (59.90, 125.24)**	**3.71 (2.92, 6.34)**
Lip and oral cavity cancer	31.63 (26.67, 37.08)	**29.84** (22.42, 37.99)	-5.64 (-15.92, 2.45)	0.47 (0.31, 0.66)	0.55 (0.35, 0.80)	17.11 (12.41, 21.12)
Nasopharynx cancer	93.03 (75.80, 111.85)	47.00 (37.31, 60.00)	-49.47 (-50.78, -46.36)	0.89 (0.59, 1.28)	0.77 (0.50, 1.17)	-13.19(-15.51, -8.66)
Other pharynx cancer				0.00 (0.00, 0.00)	0.00 (0.00, 0.00)	0.00 (0.00, 0.00)
Esophageal cancer				0.00 (0.00, 0.00)	0.00 (0.00, 0.00)	0.00 (0.00, 0.00)
Stomach cancer	118.82 (91.60, 143.21)	54.39 (42.00, 69.19)	-54.22 (-54.15, -51.68)	0.69 (0.46, 0.98)	0.40 (0.26, 0.57)	-41.75(-42.06, -1.55)
Colon and rectum cancer	117.39 (94.15, 137.55)	72.17 (58.54, 88.10)	-38.53 (-37.83, -35.95)	1.60 (1.07, 2.19)	1.62 (1.08, 2.31)	1.16 (0.71, 5.64)
Liver cancer	237.65 (180.30, 314.09)	115.91 (87.49, 158.87)	-51.23 (-51.47, -49.42)	1.68 (0.97, 2.58)	0.94 (0.58, 1.47)	-43.71 (-40.05, -43.21)
Gallbladder and biliary tract cancer	59.08 (24.96, 87.06)	27.97 (17.32, 41.67)	-52.66 (-30.63, -52.14)	0.00 (0.00, 0.00)	0.00 (0.00, 0.00)	0.00 (0.00, 0.00)
Pancreatic cancer	12.30 (10.32, 14.55)	10.91 (9.12c, 13.29)	-11.30 (-11.59, -8.67)	0.04 (0.03, 0.06)	0.04 (0.03, 0.06)	-4.66 (-5.63, -2.95)
Larynx cancer				0.00 (0.00, 0.00)	0.00 (0.00, 0.00)	0.00 (0.00, 0.00)
Tracheal, bronchus, and lung cancer	111.81 (91.15, 139.73)	79.64 (61.33, 104.94)	-28.78 (-32.71, -24.89)	0.45 (0.30, 0.64)	0.35 (0.23, 0.50)	-22.16 (-23.74, -22.01)
Malignant skin melanoma	27.70 (22.20, 33.21	12.78 (9.73, 16.31)	-53.88 (-56.20, -50.88)	1.76 (0.87, 2.96)	1.32 (0.66, 2.20)	-24.93 (-24.40, -25.79)
Non-melanoma skin cancer				0.00 (0.00, 0.00)	0.00 (0.00, 0.00)	0.00 (0.00, 0.00)
Soft tissue and other extraosseous sarcomas	167.97 (125.50, 222.22)	105.88 (79.67, 145.47)	-36.96 (-36.52, -34.54)	3.32 (2.13, 4.89)	2.84 (1.80, 4.33)	-14.42 (-15.51, -11.53)
Malignant neoplasm of bone and articular cartilage	413.90 (303.80, 553.30)	366.80 (258.83, 507.02)	-11.38 (-14.80, -8.37)	7.95 (4.96, 11.95)	8.23 (5.00, 12.52)	3.45 (0.72, 4.72)
Breast cancer	39.05 (27.94, 54.13)	47.98 (32.58, 71.16)	22.89 (16.60, 31.47)	1.06 (0.64, 1.65)	1.71 (1.02, 2.76)	61.82 (58.77, 67.30)
Cervical cancer	54.49 (40.35, 71.24)	30.34 (22.26, 43.47)	-44.32 (-44.85, -38.98)	1.39 (0.86, 2.10)	1.10 (0.68, 1.73)	-21.02 (-21.64, -17.40)
Uterine cancer				0.00 (0.00, 0.00)	0.00 (0.00, 0.00)	0.00 (0.00, 0.00)
Prostate cancer				0.00 (0.00, 0.00)	0.00 (0.00, 0.00)	0.00 (0.00, 0.00)
Ovarian cancer	45.71 (33.01, 61.03)	44.43 (32.15, 56.62)	-2.79 (-2.63, -7.21)	1.96 (1.02, 3.30)	2.42 (1.43, 3.74)	23.25 (40.49, 13.24)
Testicular cancer				1.98 (0.99, 3.35)	2.47 (1.30, 4.19)	24.90 (31.20, 25.19)
Kidney cancer	119.57 (94.15, 149.66)	72.03 (56.40, 90.69)	-39.76 (-40.09, -39.40)	1.95 (1.24, 2.88)	1.91 (1.20, 2.83)	-2.08 (-3.44, -1.86)
Bladder cancer	6.91 (5.28, 7.99)	3.88 (3.16, 4.70)	-43.89 (-40.15, -41.21)	0.26 (0.17, 0.37)	0.22 (0.15, 0.33)	-14.11 (-13.09, -12.75)
Brain and central nervous system cancer	983.40 (728.30, 1241.83)	804.52 (632.32, 1001.61)	-18.19 (-13.18, -19.34)	7.85 (5.01, 11.53)	10.46 (6.68, 15.55)	33.21 (33.40, 34.85)
Eye cancer	50.17 (24.50, 83.30)	30.96 (15.44, 55.93)	-38.29 (-37.01, -32.85)	1.26 (0.66, 2.19)	1.48 (0.72, 2.62)	18.21 (9.74, 19.67)
Neuroblastoma and other peripheral nervous cell tumors	60.89 (49.06, 75.77)	60.60 (46.71, 78.09)	-0.48 (-4.80, 3.06)	1.61 (1.00, 2.45)	1.76 (1.12, 2.70)	9.56 (12.13, 10.31)
Thyroid cancer	18.44 (14.59, 23.23)	14.20 (10.50, 19.32)	-23.02 (-28.02, -16.85)	1.34 (0.85, 2.02)	1.60 (0.95, 2.56)	19.72 (11.47, 27.08)
Mesothelioma				0.00 (0.00, 0.00)	0.00 (0.00, 0.00)	0.00 (0.00, 0.00)
Hodgkin lymphoma	139.20 (90.63, 188.21)	67.96 (47.98, 98.89)	-51.18 (-47.06, -47.45)	2.31 (1.22, 3.78)	2.13 (1.15, 3.56)	-7.87 (-6.16, -5.73)
Non-Hodgkin lymphoma	553.60 (436.46, 680.28)	329.82 (262.63, 421.55)	-40.42 (-39.83, -38.03)	8.80 (5.48, 13.06)	9.26 (5.72, 14.25)	5.23 (4.40, 9.08)
Multiple myeloma				0.00 (0.00, 0.00)	0.00 (0.00, 0.00)	0.00 (0.00, 0.00)
Leukemia	2938.62 (2213.99, 3702.35)	1520.34 (1131.74, 1952.55)	-48.26 (-48.88, -47.26)	17.59 (11.46, 25.31)	19.16 (11.84, 29.02)	8.93 (3.28, 14.66)
Other malignant neoplasms	722.76 (478.24, 952.36)	412.57 (312.37, 536.35)	-42.92 (-34.68, -43.68)	6.08 (3.45, 9.40)	6.11 (3.83, 9.41)	0.49 (10.76, 0.11)
Other neoplasms	20.31 (13.47, 33.61)	21.51 (15.05, 33.23)	5.94 (11.70, -1.14)	10.74 (5.95, 17.48)	9.32 (5.11, 15.10)	-13.27(-14.01, -13.58)

**Cardiovascular diseases**	**6389.57 (5219.96, 7744.61)**	**3728.41 (3038.92, 4538.05)**	**-41.65 (-41.78, -41.40)**	**794.10 (544.19, 1112.08)**	**785.29 (530.59, 1113.65)**	**-1.11 (-2.50, 0.14)**
Rheumatic heart disease	1902.01 (1315.19, 2582.03)	**1062.13** (756.66, 1491.19)	-44.16 (-42.47, -42.25)	307.69 (169.90, 500.16)	312.66 (170.42, 507.87)	1.62 (0.31, 1.54)
Ischemic heart disease	711.72 (587.72, 858.67)	544.94 (446.36, 661.59)	-23.43 (-24.05, -22.95)	5.15 (2.93, 8.28)	3.43 (1.56, 6.24)	-33.38 (-46.52, -24.65)
Stroke	2155.12 (1640.53, 2880.84)	1029.90 (774.63, 1336.47)	-52.21 (-52.78, -53.61)	353.06 (243.72, 470.01)	300.32 (207.71, 400.63)	-14.94 (-14.78, -14.76)
Hypertensive heart disease	55.09 (31.45, 81.02)	33.02 (21.05, 47.89)	-40.06 (-33.08, -40.90)	1.40 (0.76, 2.34)	1.46 (0.77, 2.50)	4.40 (0.19, 6.76)
Cardiomyopathy and myocarditis	826.37 (611.43, 1149.42)	537.52 (380.11, 743.56)	-34.95 (-37.83, -35.31)	57.63 (33.63, 91.80)	68.56 (39.16, 110.11)	18.97 (16.43, 19.95)
Pulmonary Arterial Hypertension	124.87 (82.55, 183.94)	72.10 (49.37, 107.84)	-42.26 (-40.20, -41.37)	0.94 (0.54, 1.49)	0.94 (0.54, 1.50)	0.00 (-0.42, 0.75)
Atrial fibrillation and flutter				0.00 (0.00, 0.00)	0.00 (0.00, 0.00)	0.00 (0.00, 0.00)
Aortic aneurysm	14.28 (10.86, 19.28)	12.63 (10.07, 16.39)	-11.50 (-7.31, -15.01)	0.00 (0.00, 0.00)	0.00 (0.00, 0.00)	0.00 (0.00, 0.00)
Lower extremity peripheral arterial disease				0.00 (0.00, 0.00)	0.00 (0.00, 0.00)	0.00 (0.00, 0.00)
Endocarditis	163.00 (99.12, 239.40)	129.45 (84.69, 197.47)	-20.58 (-14.56, -17.52)	1.96 (1.19, 3.04)	3.43 (2.03, 5.39)	74.92 (70.09, 77.43)
Non-rheumatic valvular heart disease	45.56 (30.70, 70.49)	41.09 (28.22, 59.58)	-9.82 (-8.05, -15.47)	0.08 (0.05, 0.13)	0.16 (0.09, 0.25)	88.77 (90.96, 87.91)
Other cardiovascular and circulatory diseases	391.54 (254.41, 608.36)	265.62 (185.71, 402.20)	-32.16 (-27.00, -33.89)	66.19 (35.60, 112.18)	94.33 (48.59, 166.57)	42.52 (36.46, 48.48)
**Chronic respiratory diseases**	**2465.72 (1657.89, 3175.40)**	**1325.95 (936.81, 1869.03)**	**-46.22 (-43.49, -41.14)**	**3285.49 (2027.91, 5071.06)**	**2327.86 (1390.72, 3675.75)**	**-29.15 (-31.42, -27.52)**
Chronic obstructive pulmonary disease	315.92 (216.46, 430.07)	191.03 (129.68, 274.92)	-39.53 (-40.09, -36.08)	142.26 (107.29, 179.06)	132.15 (98.24, 168.01)	-7.11 (-8.44, -6.17)
Pneumoconiosis	1.13 (0.79, 1.57)	0.38 (0.26, 0.57)	-66.24 (-67.13, -63.74)	0.24 (0.10, 0.47)	0.22 (0.09, 0.42)	-8.73 (-7.76, -10.09)
Asthma	1321.74 (886.49,1740.73)	573.04 (410.29, 809.87)	-56.65 (-53.72, -53.48)	3113.80 (1875.54, 4887.64)	2146.21 (1228.25, 3477.24)	-31.07 (-34.51, -28.86)
Interstitial lung disease and pulmonary sarcoidosis	93.84 (50.28, 163.10)	94.27 (53.75, 159.84)	0.46 (6.90, -2.00)	0.82 (0.39, 1.52)	0.97 (0.50, 1.72)	19.20 (30.48, 13.02)
Other chronic respiratory diseases	733.10 (440.49, 1052.31)	467.24 (272.51, 751.00)	-36.27 (-38.13, -28.63)	28.37 (21.40, 35.66)	48.31 (35.94, 61.35)	70.25 (67.90, 72.04)
**Digestive diseases**	**4752.03 (3756.70, 5872.95)**	**1880.91 (1490.51, 2328.44)**	**-60.42 (-60.32, -60.35)**	**878.27 (596.58, 1240.04)**	**760.01 (513.96, 1081.99)**	**-13.47 (-13.85, -12.75)**
Cirrhosis and other chronic liver diseases	1791.35(1395.33,2332.51)	843.61 (673.39, 1045.33)	-52.91 (-51.74, -55.18)	71.50 (46.32, 108.94)	49.78 (31.80, 76.93)	-30.37 (-31.35, -29.38)
Upper digestive system diseases	400.37 (290.47, 538.89)	126.52 (92.13, 172.13)	-68.40 (-68.28, -68.06)	304.53 (172.16, 491.72)	286.43 (159.06, 470.01)	-5.94 (-7.61, -4.41)
Appendicitis	375.89 (247.30, 514.75)	108.66 (75.54, 147.06)	-71.09 (-69.46, -71.43)	45.20 (21.69, 78.14)	49.13 (23.66, 86.28)	8.70 (9.08, 10.41)
Paralytic ileus and intestinal obstruction	1345.84 (1002.66, 1729.79)	534.36 (387.38, 709.61)	-60.29 (-61.37, -58.98)	14.62 (9.67, 20.13)	15.39 (10.20, 21.02)	5.31 (5.46, 4.45)
Inguinal, femoral, and abdominal hernia	282.99 (149.11, 471.08)	64.70 (39.72, 100.21)	-77.14 (-73.36, -78.73)	156.26 (90.98, 257.17)	120.86 (69.15, 198.29)	-22.65 (-23.99, -22.90)
Inflammatory bowel disease	53.18 (31.83, 78.36)	21.59 (15.49, 29.19)	-59.40 (-51.33, -62.74)	11.40 (6.63, 17.97)	11.25 (6.50, 17.32)	-1.28 (-1.90, -3.63)
Vascular intestinal disorders	16.37 (11.82, 21.85)	8.41 (6.48, 11.07)	-48.61 (-45.21, -49.35)	2.13 (0.98, 3.87)	2.21 (1.05, 3.95)	3.61 (6.74, 1.97)
Gallbladder and biliary diseases	59.08 (24.96, 87.06)	27.97 (17.32, 41.67)	-52.66 (-30.63, -52.14)	218.15 (125.94, 347.99)	182.81 (104.12, 294.20)	-16.20 (-17.33, -15.46)
Pancreatitis	84.03 (56.80, 128.27)	52.48 (36.78, 75.92)	-37.55 (-35.24, -40.81)	14.54 (6.65, 27.57)	13.40 (6.29, 24.81)	-7.82 (-5.43, -10.02)
Other digestive diseases	342.95 (219.48, 478.40)	92.61 (65.01, 135.07)	-73.00 (-70.38, -71.77)	39.95 (26.64, 56.48)	28.74 (19.17, 41.02)	-28.06(-28.04, -27.36)
**Neurological disorders**	**1747.64 (1362.81, 2078.99)**	**1348.27 (1087.75, 1662.20)**	**-22.85 (-20.18, -20.05)**	**7243.67 (2164.01, 15325.65)**	**7295.58 (2111.88, 15498.35)**	**0.72 (-2.41, 1.13)**
Alzheimer's disease and other dementias				0.00 (0.00, 0.00)	0.00 (0.00, 0.00)	0.00 (0.00, 0.00)
Parkinson's disease				0.00 (0.00, 0.00)	0.00 (0.00, 0.00)	0.00 (0.00, 0.00)

Idiopathic epilepsy	1408.67 (1045.96, 1724.41)	1011.78 (777.46, 1290.98)	-28.17 (-25.67, -25.14)	1423.46 (681.24, 2509.48)	1268.15 (586.68, 2345.38)	-10.91 (-13.88, -6.54)
Multiple sclerosis	2.13 (1.61, 2.82)	1.64 (1.22, 2.18)	-22.79 (-24.14, -22.55)	3.69 (1.90, 6.11)	4.06 (2.10, 6.78)	10.05 (10.67, 11.07)
Motor neuron disease	54.11 (41.70, 64.76)	30.98 (22.33, 40.20)	-42.74 (-46.45, -37.93)	6.18 (3.95, 9.18)	6.41 (4.10, 9.56)	3.78 (3.72, 4.14)
Headache disorders				5578.64 (595.85, 13346.20)	5640.63 (595.86, 13474.86)	1.11 (0.00, 0.96)
Other neurological disorders	282.73 (243.13, 334.83)	303.86 (245.45, 367.74)	7.47 (0.95, 9.83)	231.71 (112.57, 405.11)	376.32 (175.87, 694.87)	62.41 (56.23, 71.53)
**Mental disorders**	**3.02 (2.14, 4.19)**	**3.89 (2.66, 5.70)**	**29.16 (24.17, 35.94)**	**17750.10 (12825.37, 23510.53)**	**19537.66 (14033.65, 26085.25)**	**10.07 (9.42, 10.95)**
Schizophrenia				380.32 (216.28, 606.86)	390.57 (224.10, 624.37)	2.69 (3.62, 2.89)
Depressive disorders				4020.52 (2507.50, 6040.02)	4714.38 (2856.48, 7226.49)	17.26 (13.92, 19.64)
Bipolar disorder				857.34 (491.45, 1399.29)	859.97 (492.43, 1392.71)	0.31 (0.20, -0.47)
Anxiety disorders				4965.16 (3032.82, 7499.90)	5852.03 (3509.35, 8967.74)	17.86 (15.71, 19.57)
Eating disorders	3.02 (2.14, 4.19)	3.89 (2.66, 5.70)	29.16 (24.17, 35.94)	786.12 (436.23, 1332.93)	991.40 (567.25, 1668.58)	26.11 (30.03, 25.18)
Autism spectrum disorders				2521.90 (1724.61, 3537.78)	2595.71 (1773.60, 3649.42)	2.93 (2.84, 3.16)
Attention-deficit/hyperactivity disorder				429.14 (221.53, 728.87)	449.70 (234.35, 747.88)	4.79 (5.79, 2.61)
Conduct disorder				2718.90 (1447.69, 4312.38)	2801.20 (1478.96, 4479.04)	3.03 (2.16, 3.86)
Idiopathic developmental intellectual disability				809.67 (359.08, 1416.30)	620.75 (274.29, 1088.31)	-23.33 (-23.61, -23.16)
Other mental disorders				261.02 (143.70, 419.35)	261.95 (144.24, 420.26)	0.35 (0.38, 0.22)
**Substance use disorders**	**526.60 (440.77, 615.70)**	**280.14 (219.47, 352.35)**	**-46.80 (-50.21, -42.77)**	**1322.43 (866.42, 1902.17)**	**1118.98 (726.76, 1612.57)**	**-15.38 (-16.12, -15.22)**
Alcohol use disorders	215.48 (175.03, 255.63)	89.06 (59.81, 121.10)	-58.67 (-65.83, -52.63)	558.38 (311.15, 912.37)	476.90 (261.58, 786.60)	-14.59 (-15.93, -13.79)
Drug use disorders	311.12 (256.72, 374.76)	**191.08** (146.80, 245.95)	-38.58 (-42.82, -34.37)	764.05 (485.75, 1104.15)	642.08 (398.59, 940.12)	-15.96 (-17.94, -14.86)
**Diabetes and kidney diseases**	**1695.38 (1265.79, 2059.75)**	**1298.61 (1026.04, 1571.41)**	**-23.40 (-18.94, -23.71)**	**392.51 (255.85, 575.05)**	**526.75 (344.08, 783.91)**	**34.20 (34.49, 36.32)**
Diabetes mellitus	440.41 (334.58, 560.69)	371.98 (290.25, 470.22)	-15.54 (-13.25, -16.14)	203.62 (123.57, 320.60)	355.35 (217.42, 557.65)	74.52 (75.95, 73.94)
Chronic kidney disease	1153.77 (808.39, 1424.44)	902.03 (687.18, 1119.49)	-21.82 (-14.99, -21.41)	188.33 (110.07, 294.67)	170.94 (99.03, 275.65)	-9.24 (-10.03, -6.45)
Acute glomerulonephritis	101.20 (63.58, 145.10)	24.60 (14.32, 35.36)	-75.69 (-77.48, -75.63)	0.56 (0.27, 1.01)	0.46 (0.23, 0.82)	-18.17 (-15.15, -19.13)
**Skin and subcutaneous diseases**	**308.29 (218.69, 425.28)**	**167.80 (115.14, 245.32)**	**-45.57 (-47.35, -42.32)**	**9702.75 (6061.58, 14547.22)**	**10155.06 (6363.41, 15245.97)**	**4.66 (4.98, 4.80)**
Dermatitis				2717.40 (1434.14, 4548.02)	2726.08 (1431.42, 4570.51)	0.32 (-0.19, 0.49)
Psoriasis				255.34 (175.89, 353.84)	294.23 (201.85, 407.77)	15.23 (14.76, 15.24)
Bacterial skin diseases	245.86 (162.29, 345.50)	135.04 (79.13, 206.50)	-45.07 (-51.24, -40.23)	35.76 (19.09, 62.91)	36.37 (19.42, 64.16)	1.71 (1.69, 1.98)
Scabies				1386.16 (711.31, 2333.45)	1388.29 (708.76, 2349.49)	0.15 (-0.36, 0.69)
Fungal skin diseases				395.69 (152.80, 847.12)	382.81 (147.99, 817.94)	-3.25 (-3.14, -3.44)
Viral skin diseases				1324.87 (831.25, 2026.91)	1402.01 (876.38, 2149.34)	5.82 (5.43, 6.04)
Acne vulgaris				2296.06 (1403.86, 3695.83)	2584.55 (1568.81, 4158.27)	12.56 (11.75, 12.51)
Alopecia areata				60.31 (37.52, 89.79)	60.62 (37.73, 90.39)	0.53 (0.56, 0.67)
Pruritus				78.21 (34.98, 156.38)	85.14 (38.03, 171.36)	8.86 (8.72, 9.57)
Urticaria				898.75 (546.31, 1384.72)	896.34 (542.81, 1383.72)	-0.27 (-0.64, -0.07)
Decubitus ulcer	17.61 (8.38, 34.40)	13.24 (5.03, 27.67)	-24.84 (-39.93, -19.56)	2.78 (1.55, 4.37)	2.89 (1.61, 4.51)	3.79 (3.86, 3.28)
Other skin and subcutaneous diseases	44.82 (21.71, 86.64)	19.52 (11.92, 37.64)	-56.44 (-45.09, -56.56)	251.43 (117.84, 462.97)	295.74 (137.93, 545.31)	17.62 (17.06, 17.79)
**Sense organ diseases**				**2266.91**	**2317.71**	**2.24**

				**(1500.89, 3257.17)**	**(1534.71, 3332.76)**	**(2.25, 2.32)**
Blindness and vision loss				777.27 (476.67, 1221.13)	748.43 (456.02, 1189.57)	-3.71 (-4.33, -2.58)
Age-related and other hearing loss				1312.79 (823.91, 1969.09)	1386.40 (871.36, 2064.84)	5.61 (5.76, 4.86)
Other sense organ diseases				176.85 (97.36, 291.43)	182.87 (100.20, 301.40)	3.40 (2.92, 3.42)
**Musculoskeletal disorders**	**212.54 (150.36, 284.55)**	**162.63 (105.09, 228.12)**	**-23.48 (-30.11, -19.83)**	**5937.57 (3819.01, 8549.81)**	**5833.32 (3742.87, 8369.99)**	**-1.76 (-1.99, -2.10)**
Rheumatoid arthritis	6.91 (5.29, 9.58)	3.13 (2.26, 4.21)	-54.68 (-57.22, -56.11)	25.30 (13.29, 42.80)	32.56 (17.65, 54.23)	28.73 (32.75, 26.70)
Osteoarthritis				0.00 (0.00, 0.00)	0.00 (0.00, 0.00)	0.00 (0.00, 0.00)
Low back pain				4214.42 (2588.43, 6254.46)	3940.74 (2401.55, 5840.50)	-6.49 (-7.22, -6.62)
Neck pain				826.48 (382.65, 1546.41)	833.03 (384.49, 1560.40)	0.79 (0.48, 0.91)
Gout				0.91 (0.27, 2.04)	1.02 (0.31, 2.28)	11.29 (14.97, 11.80)
Other musculoskeletal disorders	205.63 (144.42, 276.56)	159.50 (102.39, 224.62)	-22.43 (-29.10, -18.78)	870.46 (480.65, 1445.20)	1025.97 (580.88, 1678.73)	17.86 (20.85, 16.16)
**Other non-communicable diseases**	**28272.95 (19384.64, 35040.07)**	**14584.60 (10126.88, 18962.14)**	**-48.42 (-47.76, -45.88)**	**6129.39 (4254.97, 8693.83)**	**5742.60 (3976.16, 8181.80)**	**-6.31 (-6.55, -5.89)**
Congenital birth defects	23978.95 (15534.42, 30472.69)	12814.10 (8629.17, 16919.71)	-46.56 (-44.45, -44.48)	2030.77 (1428.98, 2805.26)	1975.30 (1389.18, 2716.64)	-2.73 (-2.79, -3.16)
Urinary diseases and male infertility	365.04 (269.34, 451.71)	258.75 (196.66, 331.36)	-29.12 (-26.98, -26.64)	46.84 (27.54, 79.69)	47.33 (27.99, 79.30)	1.04 (1.63, -0.50)
Gynecological diseases	16.96 (9.10, 29.28)	17.43 (9.28, 28.09)	2.73 (1.89, -4.05)	1458.71 (853.32, 2417.68)	1461.46 (857.92, 2434.55)	0.19 (0.54, 0.70)
Hemoglobinopathies and hemolytic anemias	704.79 (494.10, 917.20)	349.00 (239.16, 466.52)	-50.48 (-51.60, -49.14)	1156.41 (740.63, 1722.12)	863.42 (542.68, 1304.50)	-25.34 (-26.73, -24.25)
Endocrine, metabolic, blood, and immune disorders	675.60 (472.80, 899.15)	502.78 (371.83, 669.75)	-25.58 (-21.36, -25.51)	483.51 (255.24, 859.61)	452.31 (237.90, 810.46)	-6.45 (-6.80, -5.72)
Oral disorders				953.15 (517.93, 1591.77)	942.79 (515.17, 1572.50)	-1.09 (-0.53, -1.21)
Sudden infant death syndrome	2531.6(1638.08, 3767.77)	642.54 (341.83,1014.50)	-74.62(-79.13, -73.1)			

Note: Data in parentheses are 95% uncertainty intervals. YLLs, years of life lost; YDLs, years of lived with disability (YLDs)

Figure S1. All-cause death rate per 100000 population due to NCDs in the Asia-Pacific region from 1990 to 2021 by age group and sex.

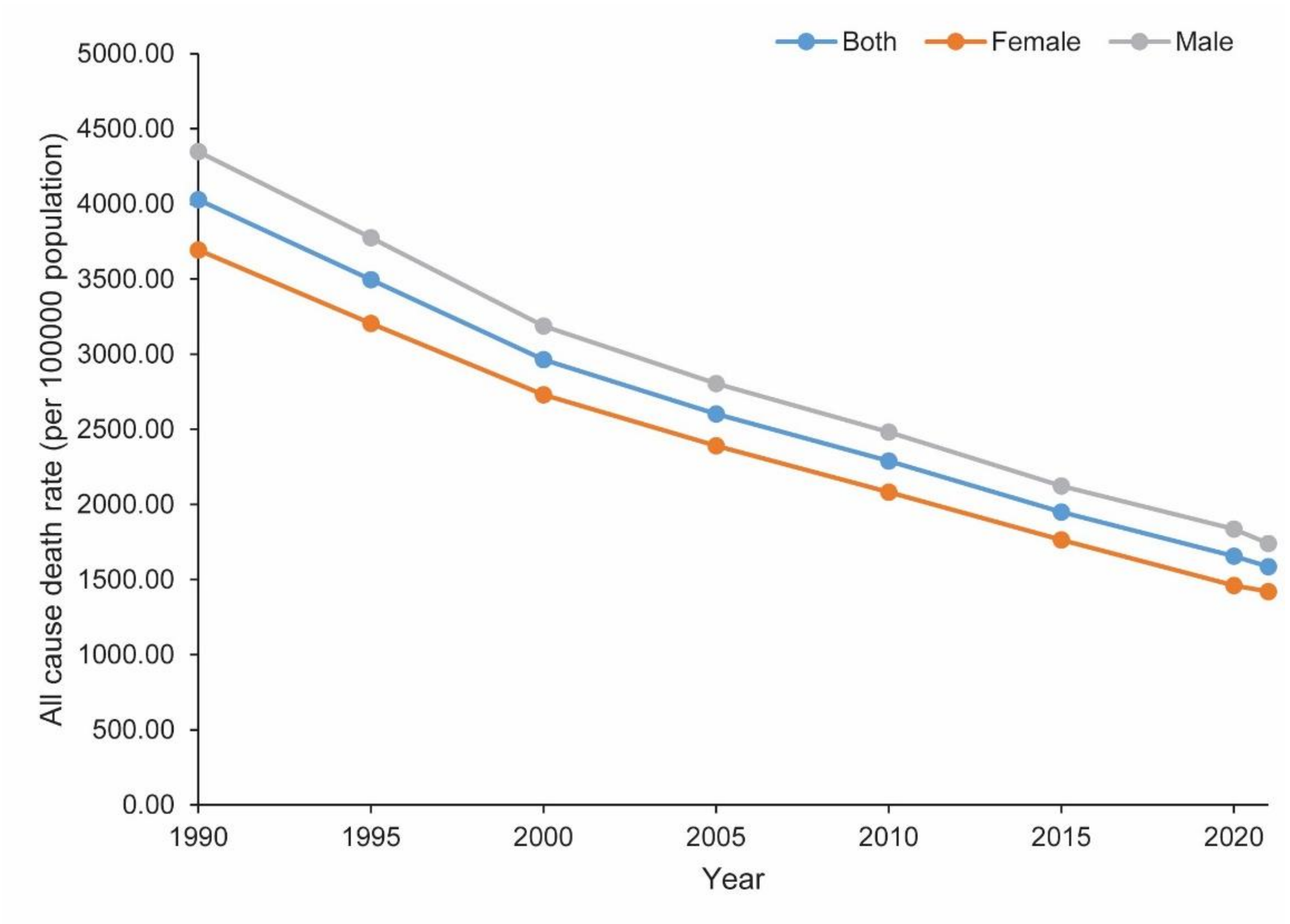

Figure S2. All-cause death rate per 100000 population due to NCDs in the Asia-Pacific region from 1990 to 2021 by age group and sex. A: Both sexes. B: Female. C: Male.

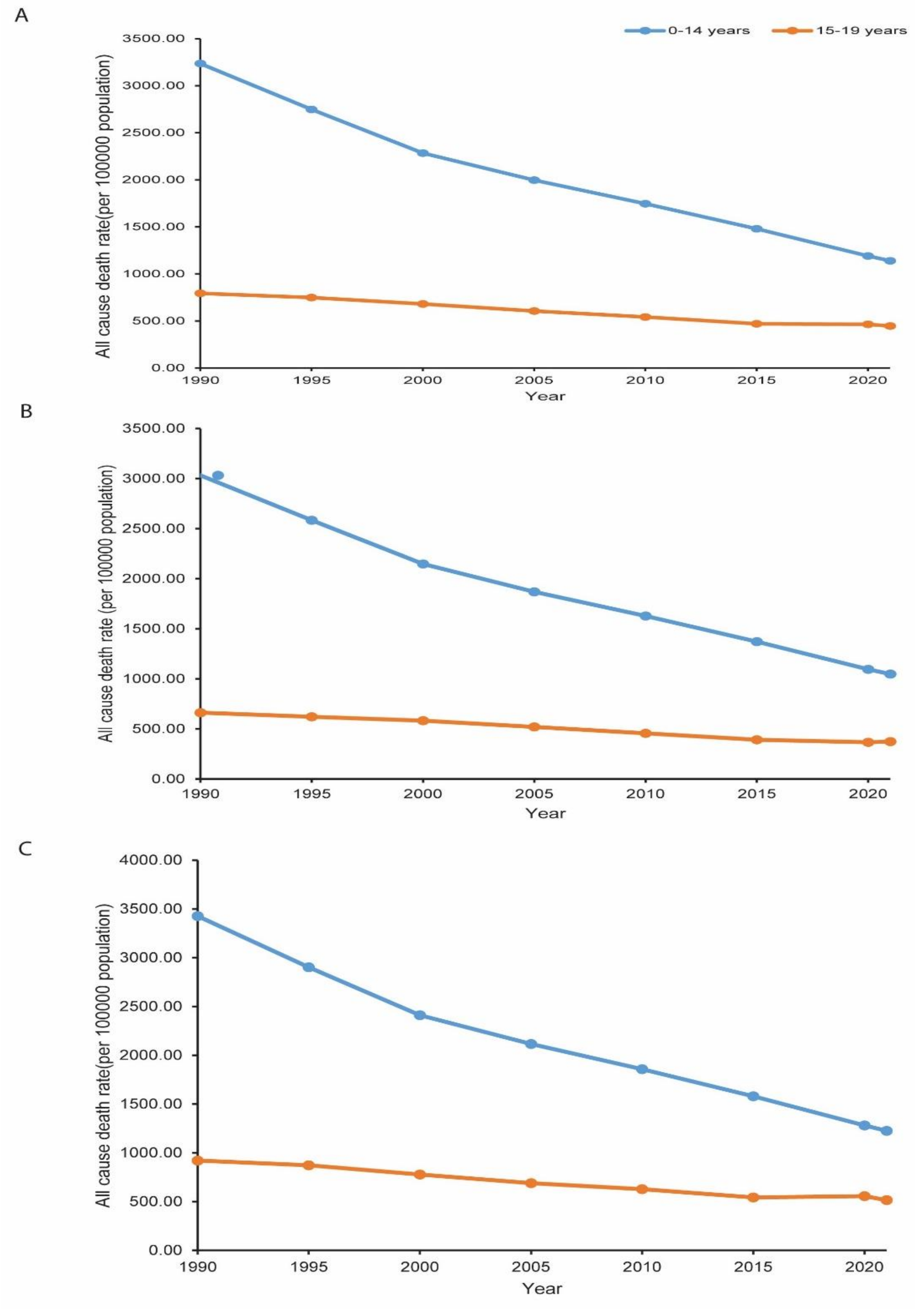

Figure S3. Death rate per 100000 population due to NCDs in the Asia-Pacific region in 2021 by sex and age group. A: 0-14 years.B: 15-19 years.

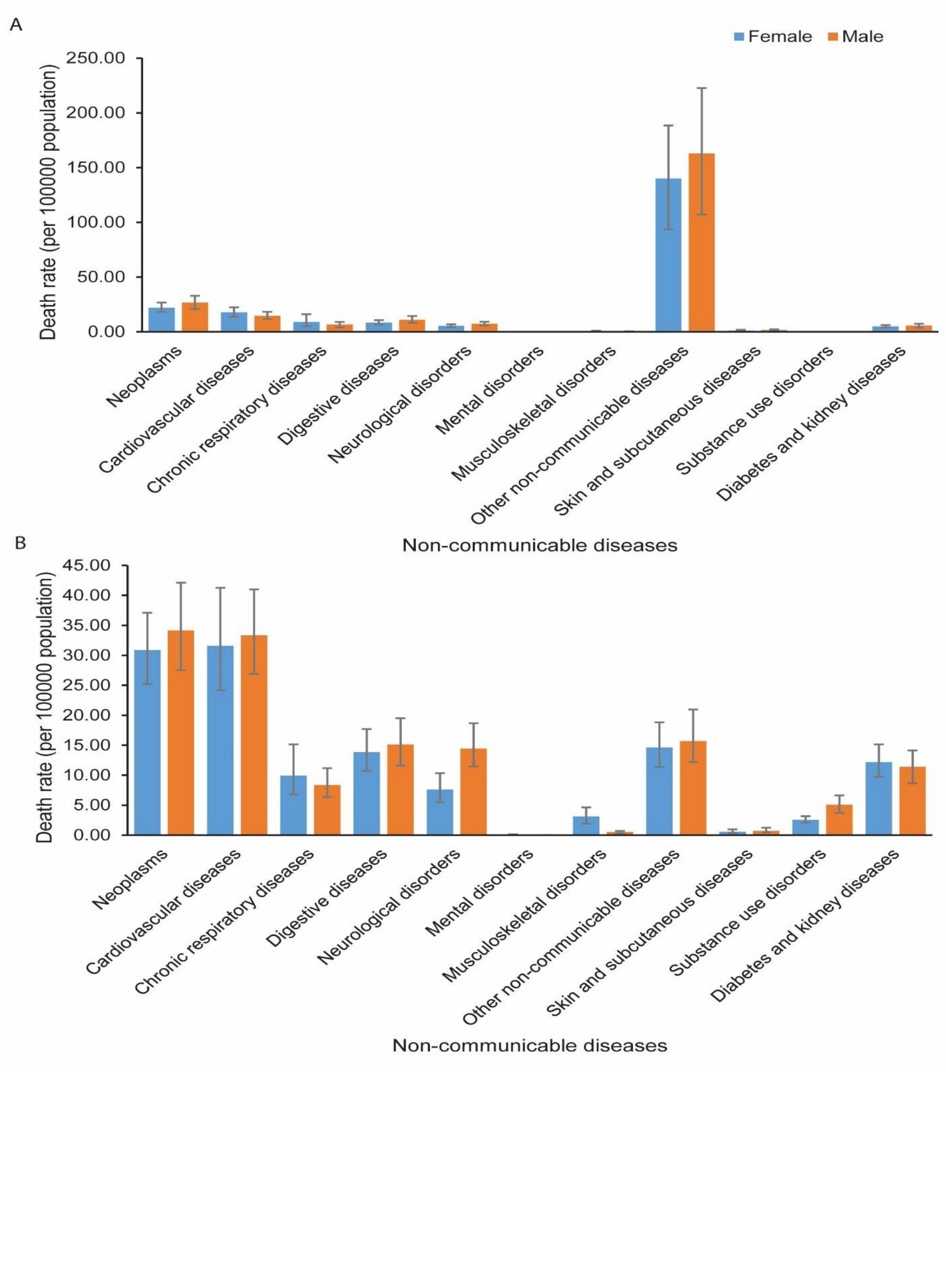

Figure S4. Death rate per 100000 population due to NCDs in the Asia-Pacific region from 1990 to 2021

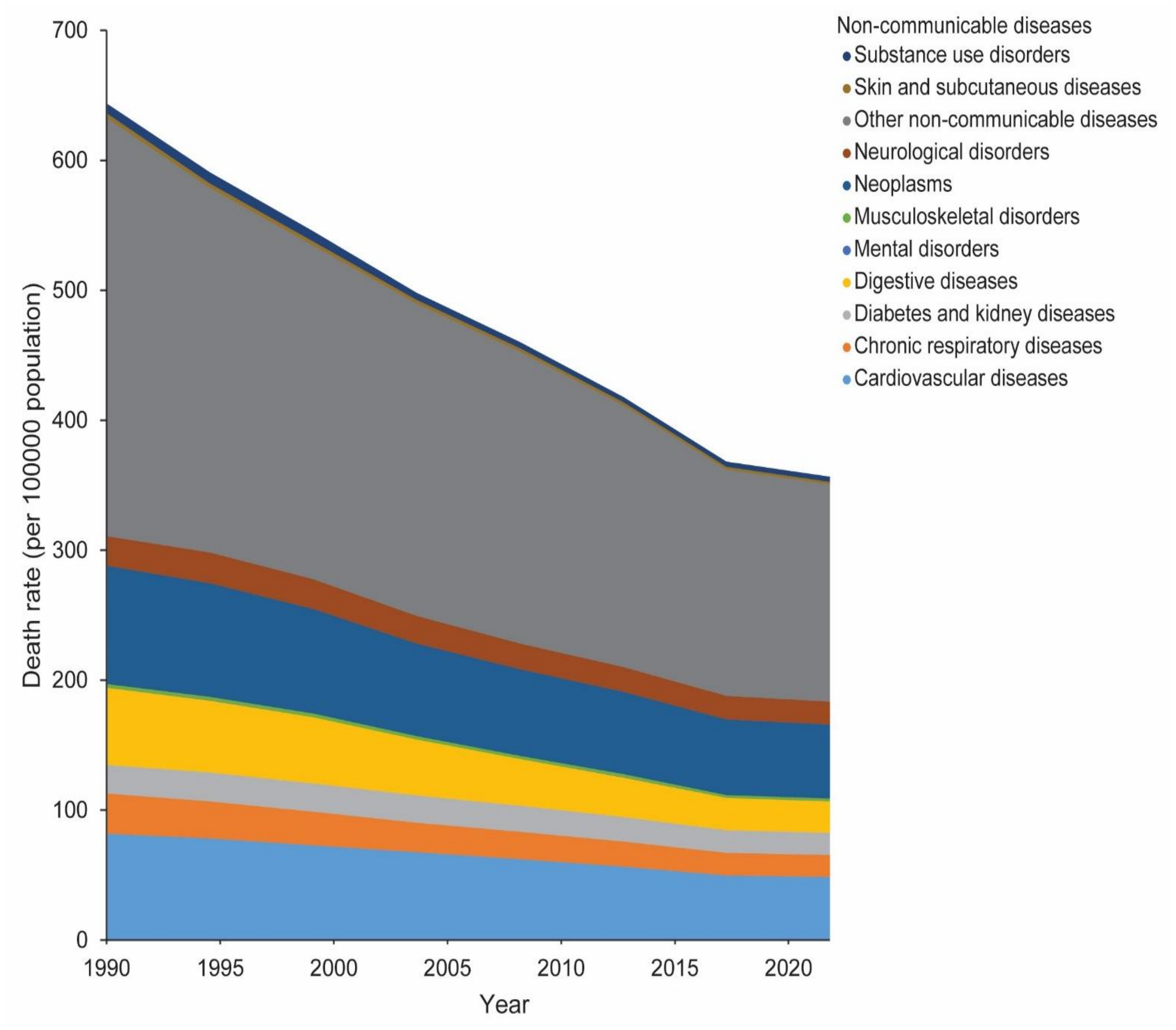

Figure S5. All-cause YLL rate per 100000 population aged 0-19 years from 1990 to 2021 by sex and age group

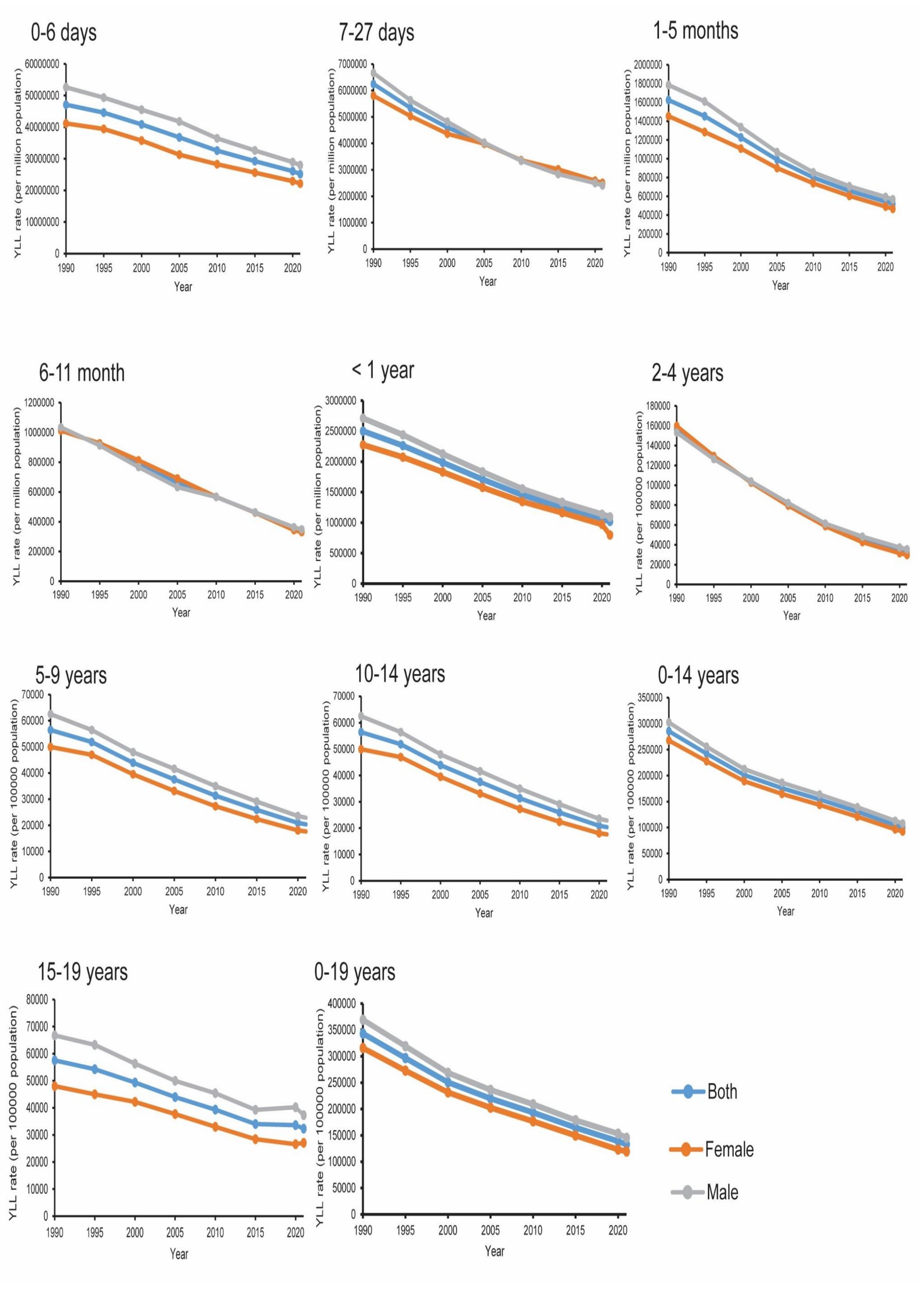

Figure S6. YLL rate per 1000000 population due to NCDs in Asia-Pacific region in 2021 by age group and sex. A: 0-14 years. B: 15-19 years.

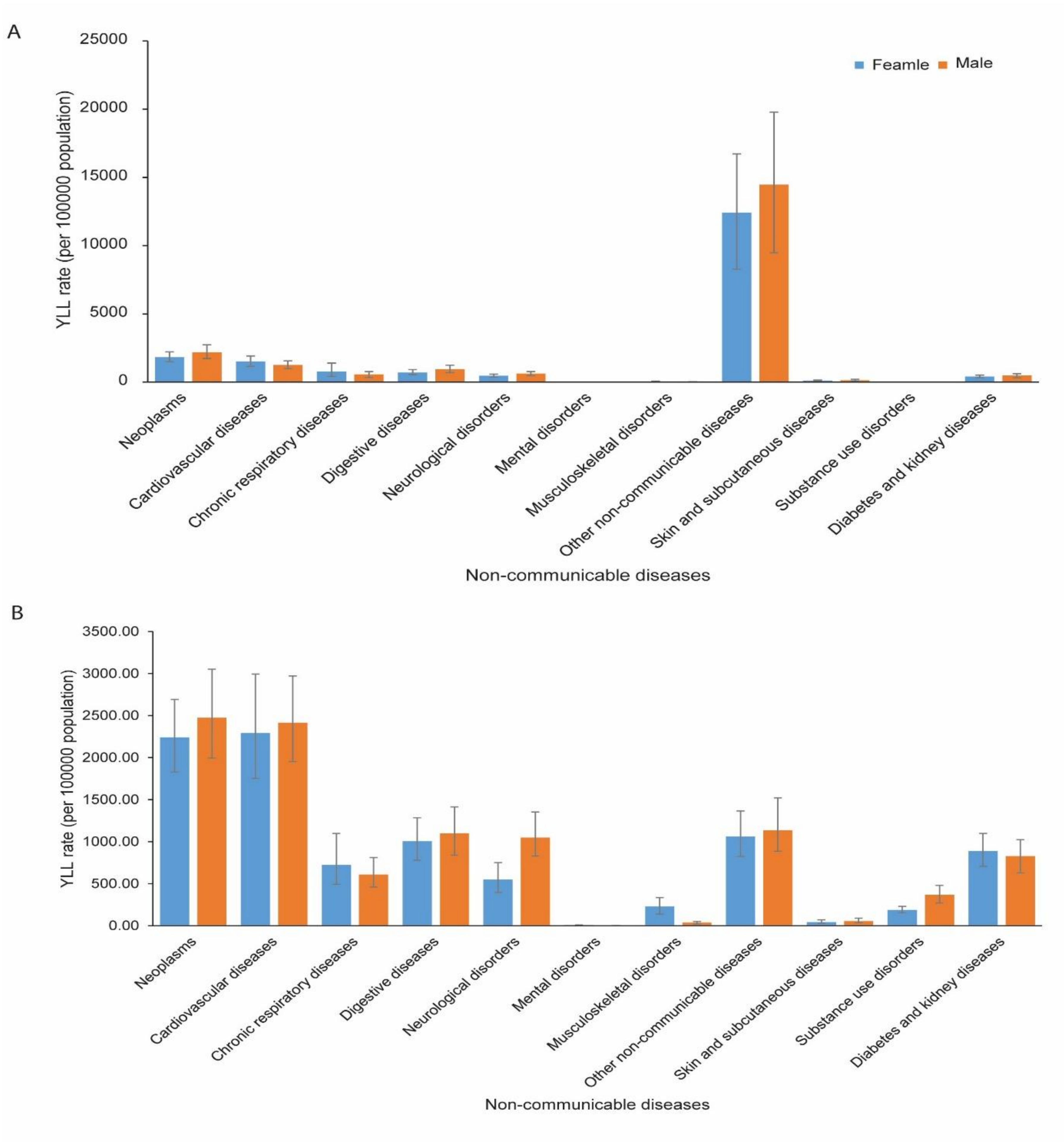

Figure S7. YLL rate per 100000 population due to the first 20 level 3 NCDs among people aged 0-19 years old in the Asia-Pacific region in 2021 by sex.

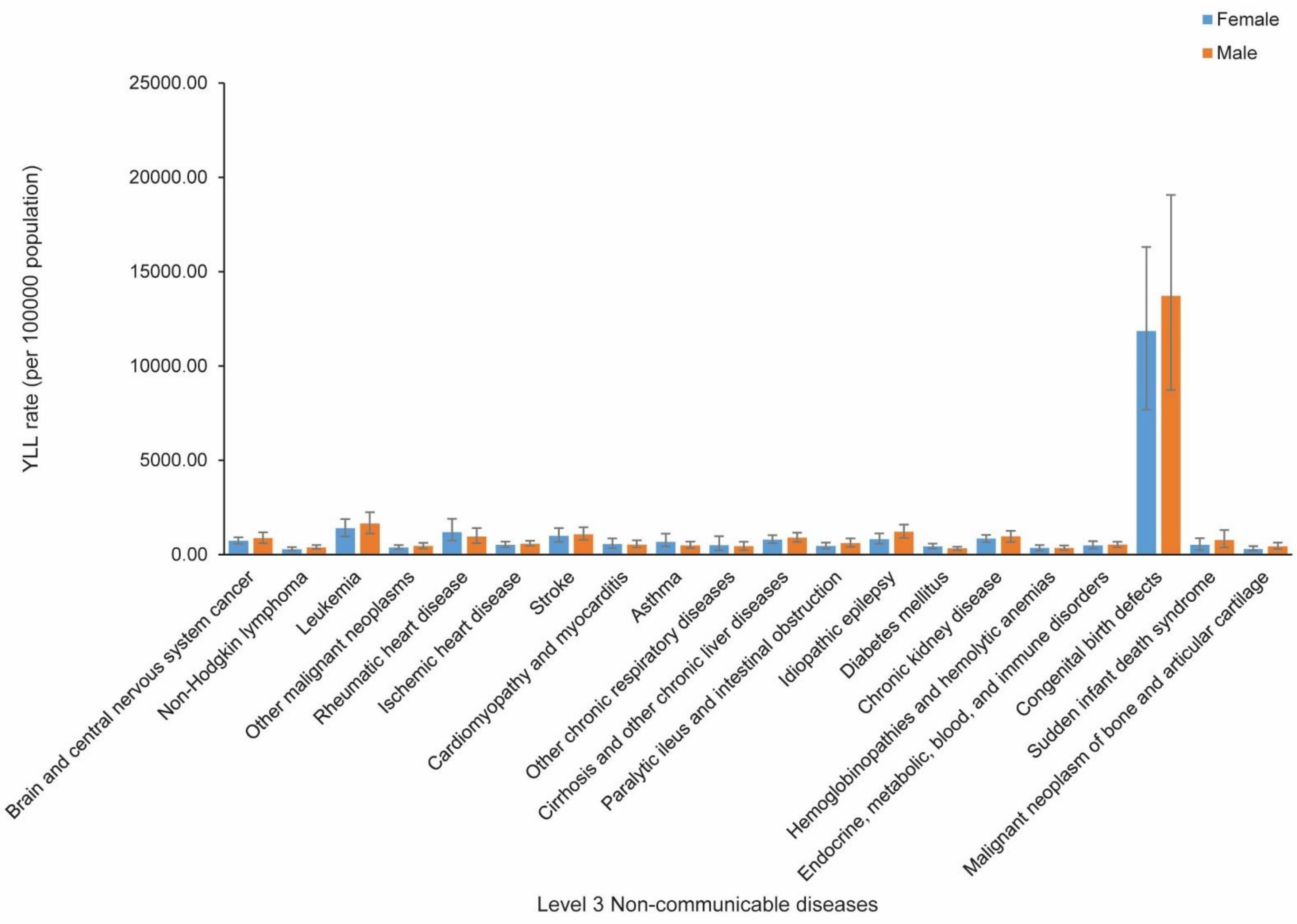

Figure S8. YLL rate per 100000 population due to NCDs among people aged 0-19 years old in the Asia-Pacific region in 2021 in both sexes by location.

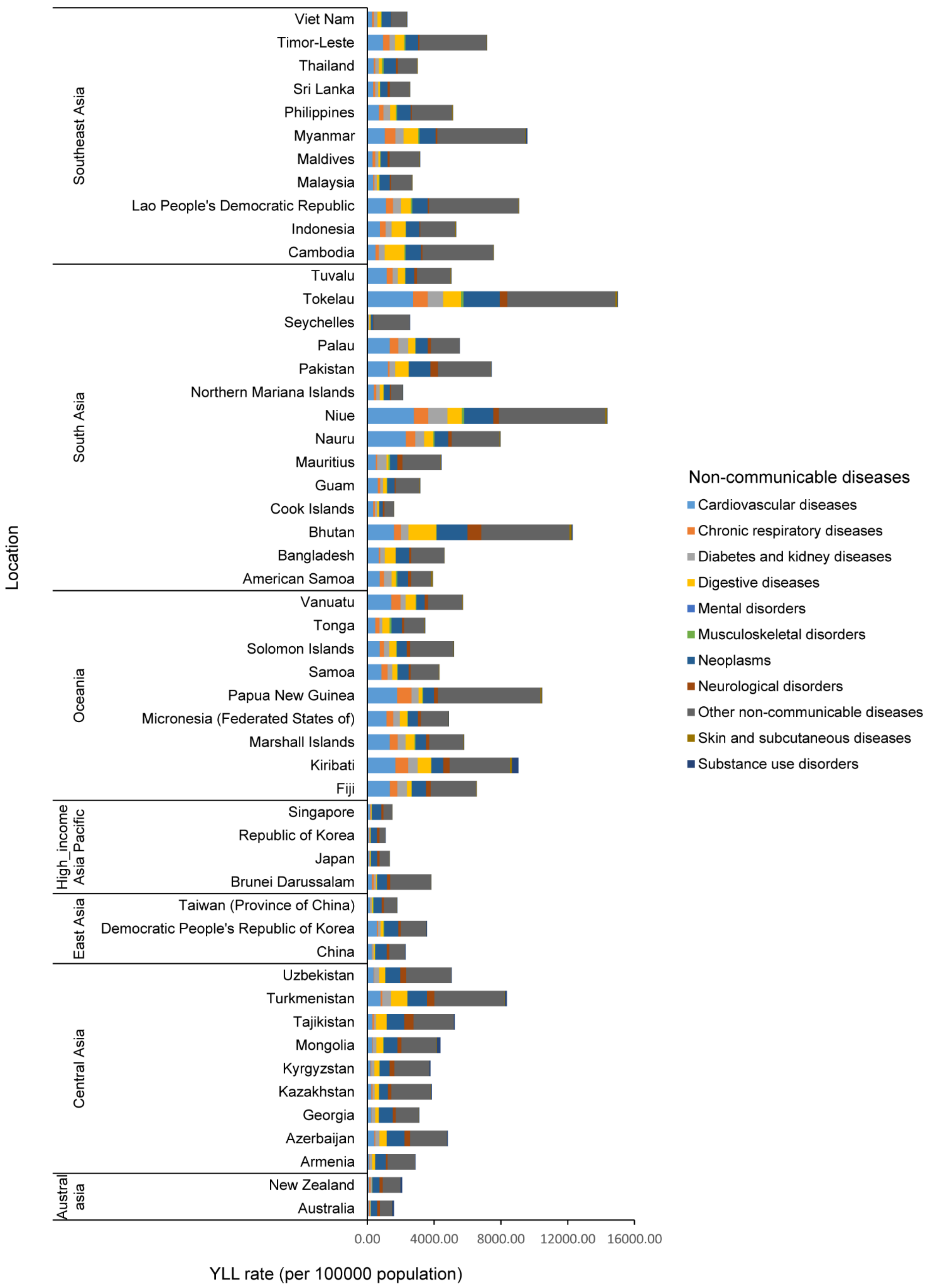

Figure S9. YLL rate per 100,000 people due to NCDs among people aged 0-19 years in the Asia-Pacific region in both sexes from 1990 to 2021.

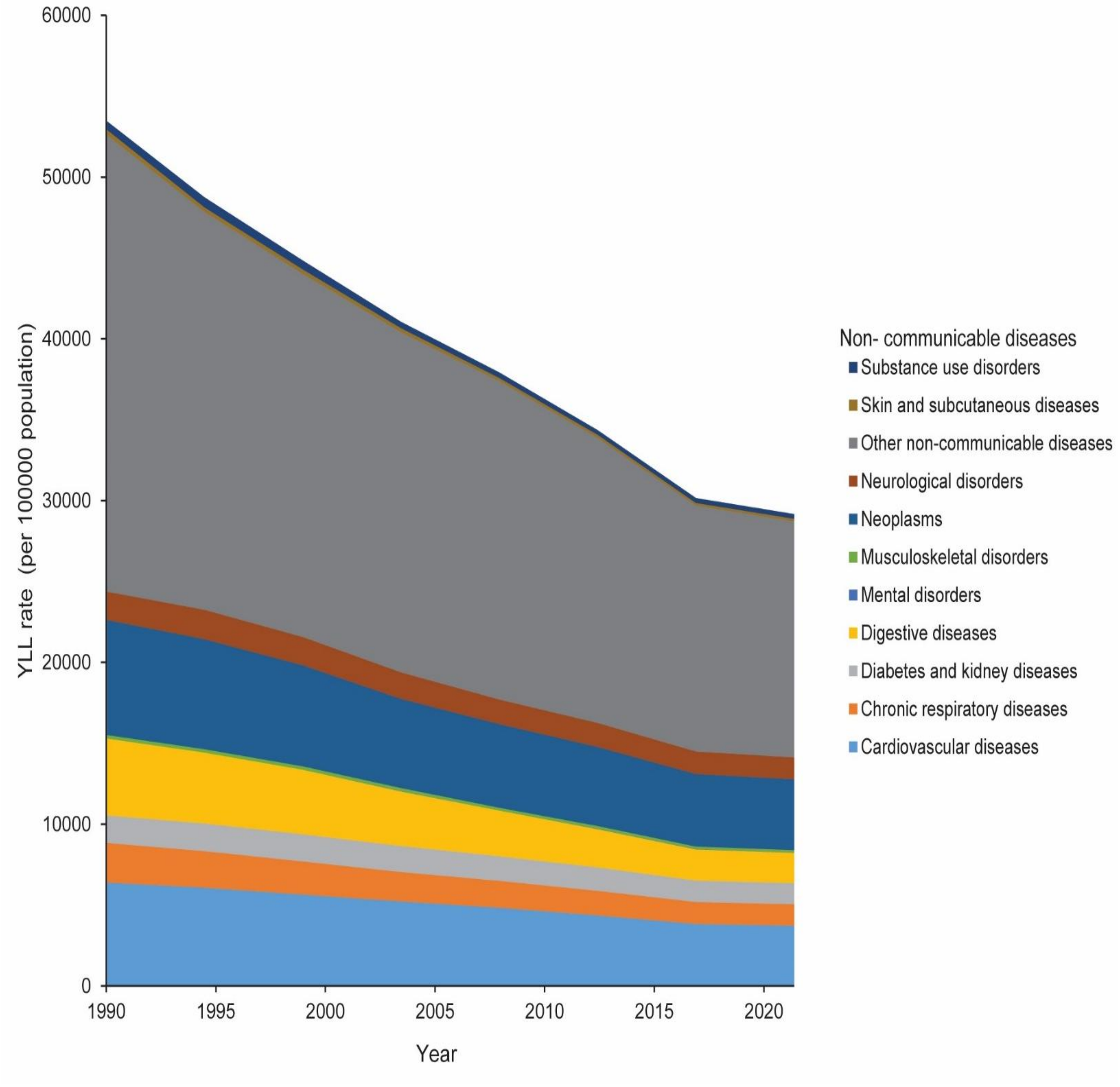

Figure S10. All-cause YLD rate per 100000 population among people aged 0-19 years from 1990-2021 by sex and age group.

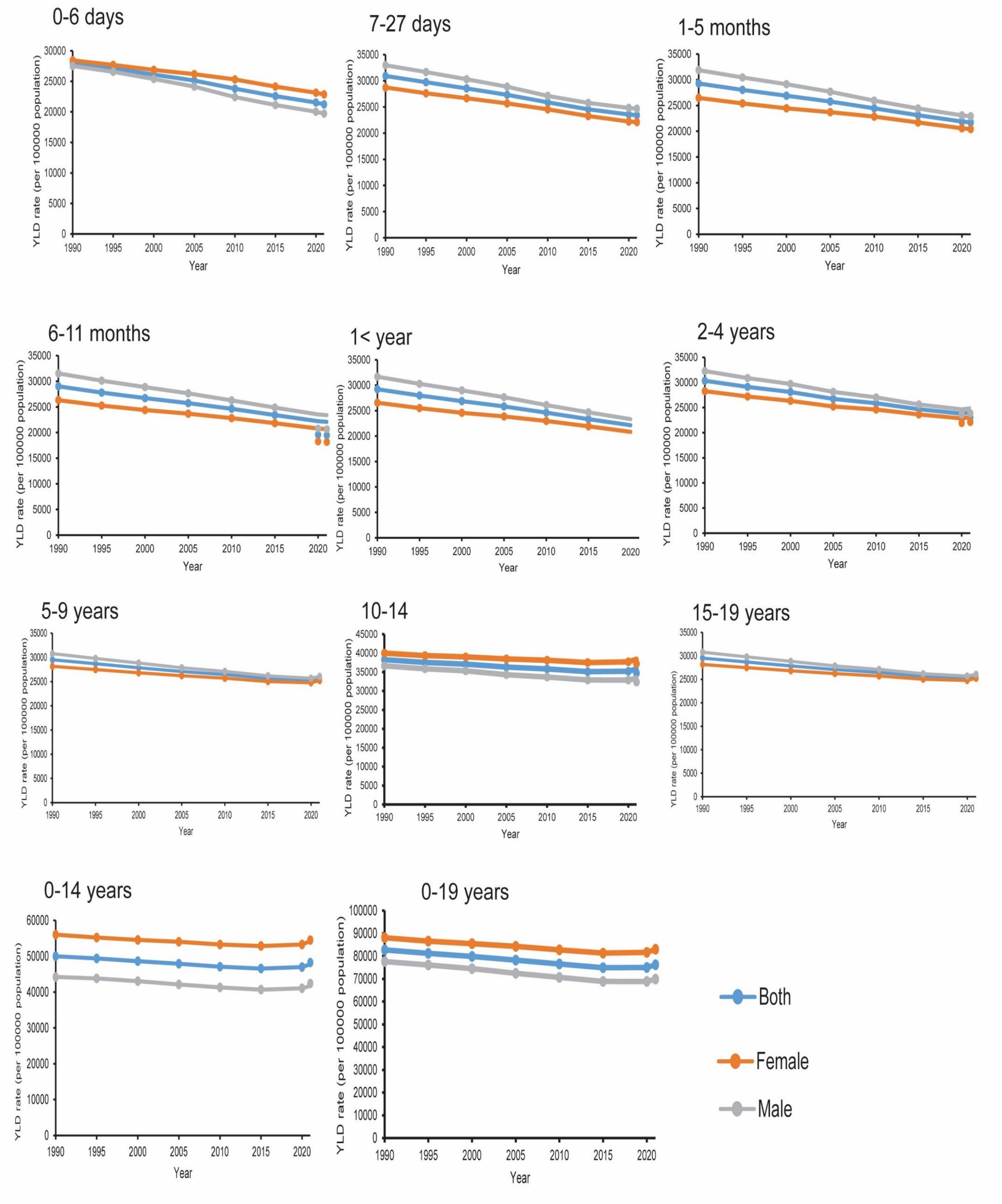

Figure S11. YLD rate per 100000 population due to NCDs among people aged 0-19 years in the Asia-Pacific region in 2021 by sex and age group. A: 0-14 years. B: 15-19 years

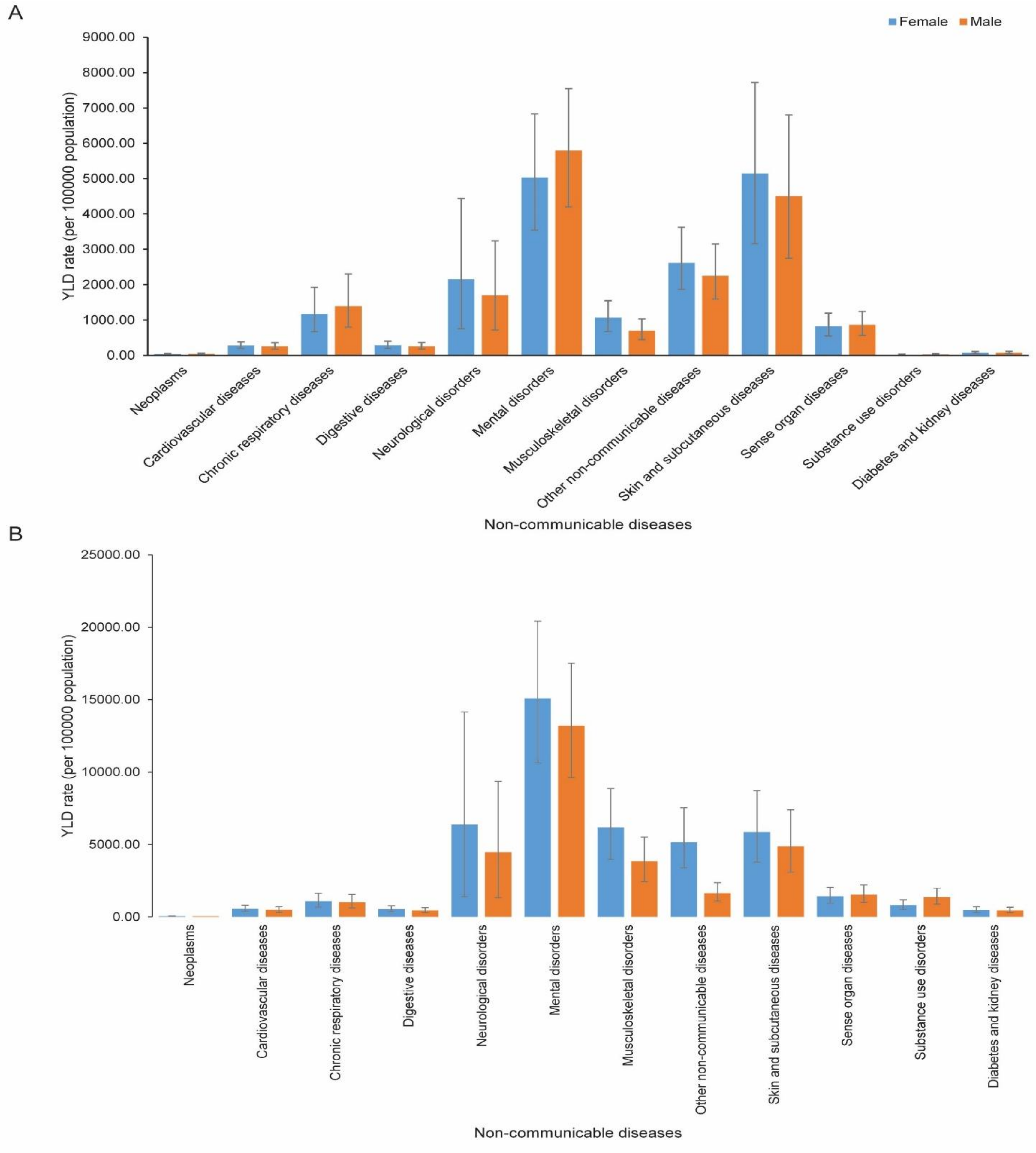

Figure S12. YLD rate per 100000 population due to the first 20 level 3 NCDs among people aged 0-19 years old in the Asia-Pacific region in 2021 by sex.

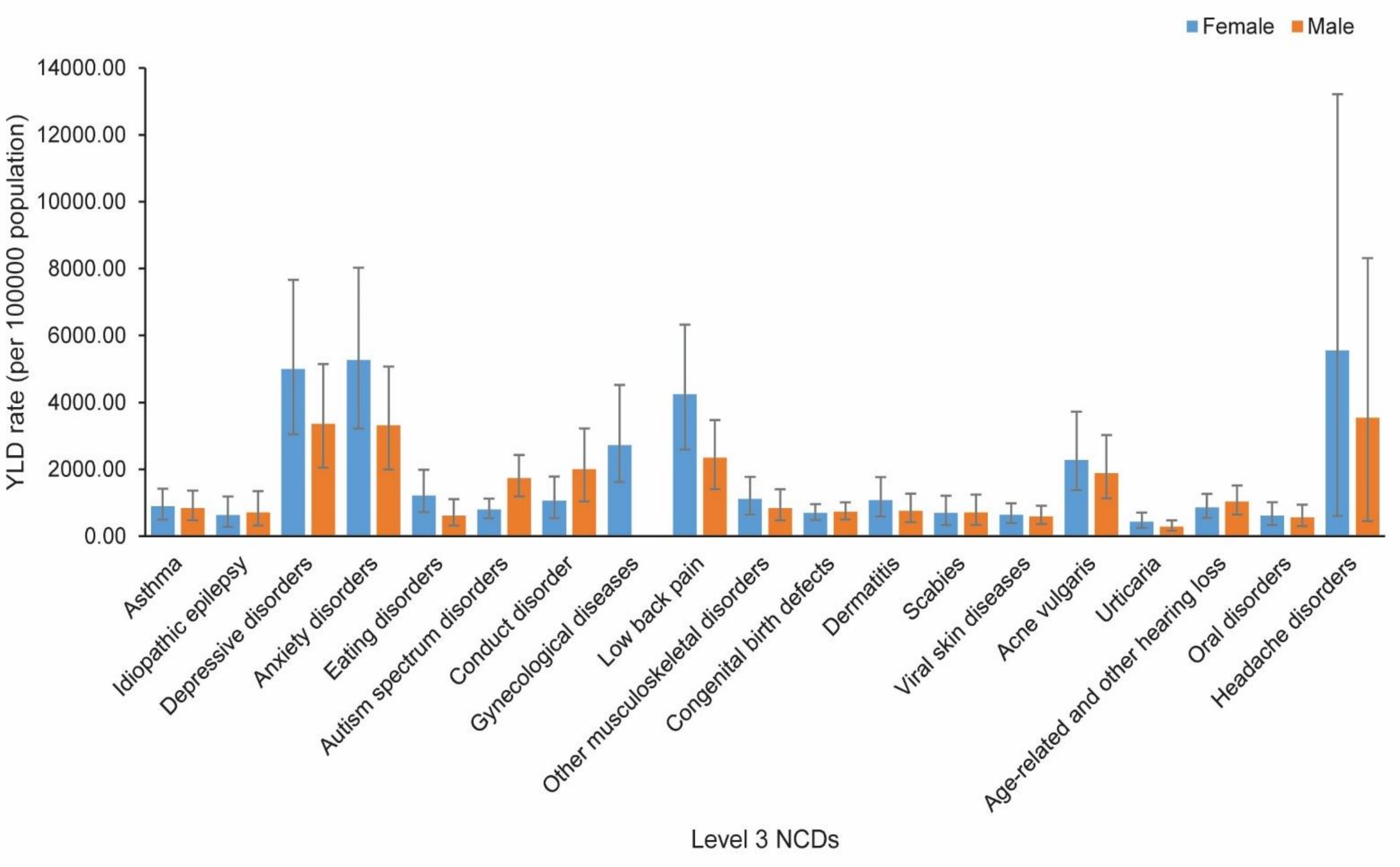

Figure S13. YLD rate per 100000 population due to NCDs among people aged 0-19 years old in the Asia-Pacific region in 2021 in both sexes by location.

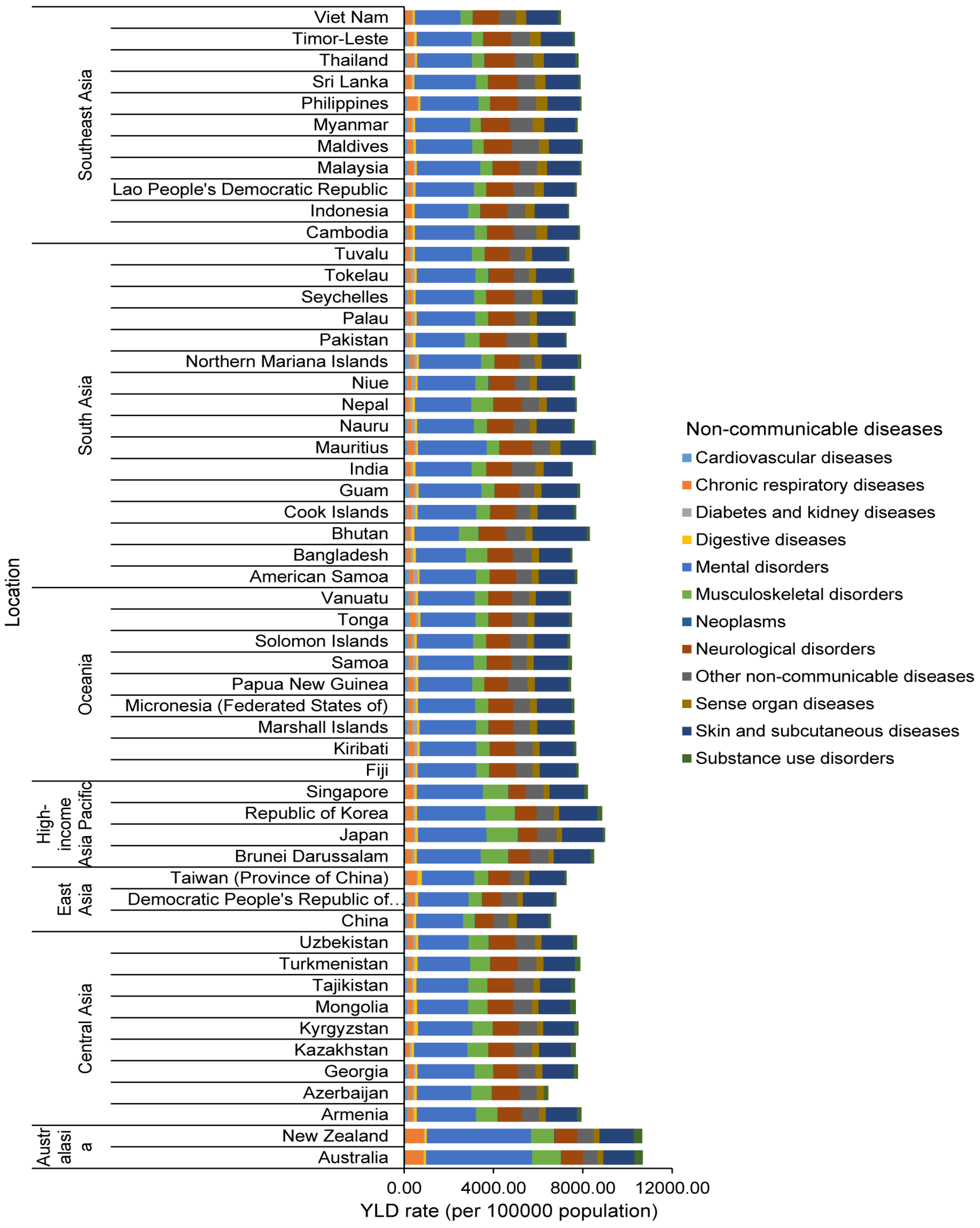

Figure S14. YLD rate per 100,000 people due to NCDs among people aged 0-19 years in the Asia-Pacific region in both sexes from 1990 to 2021.

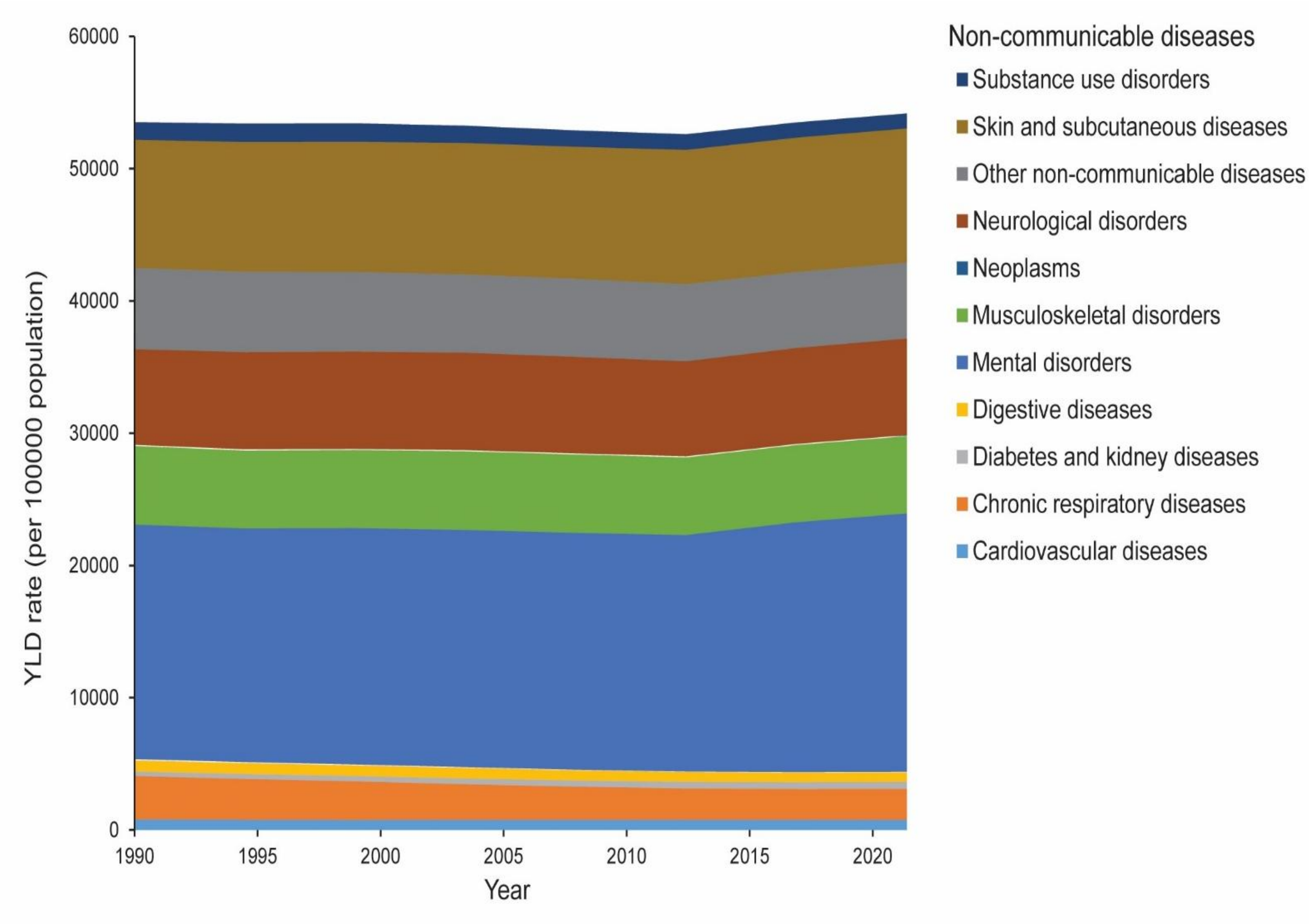

Figure S15. All-cause DALY rate per 100000 population among people aged 0-19 years from 1990-2021 by sex and age group.

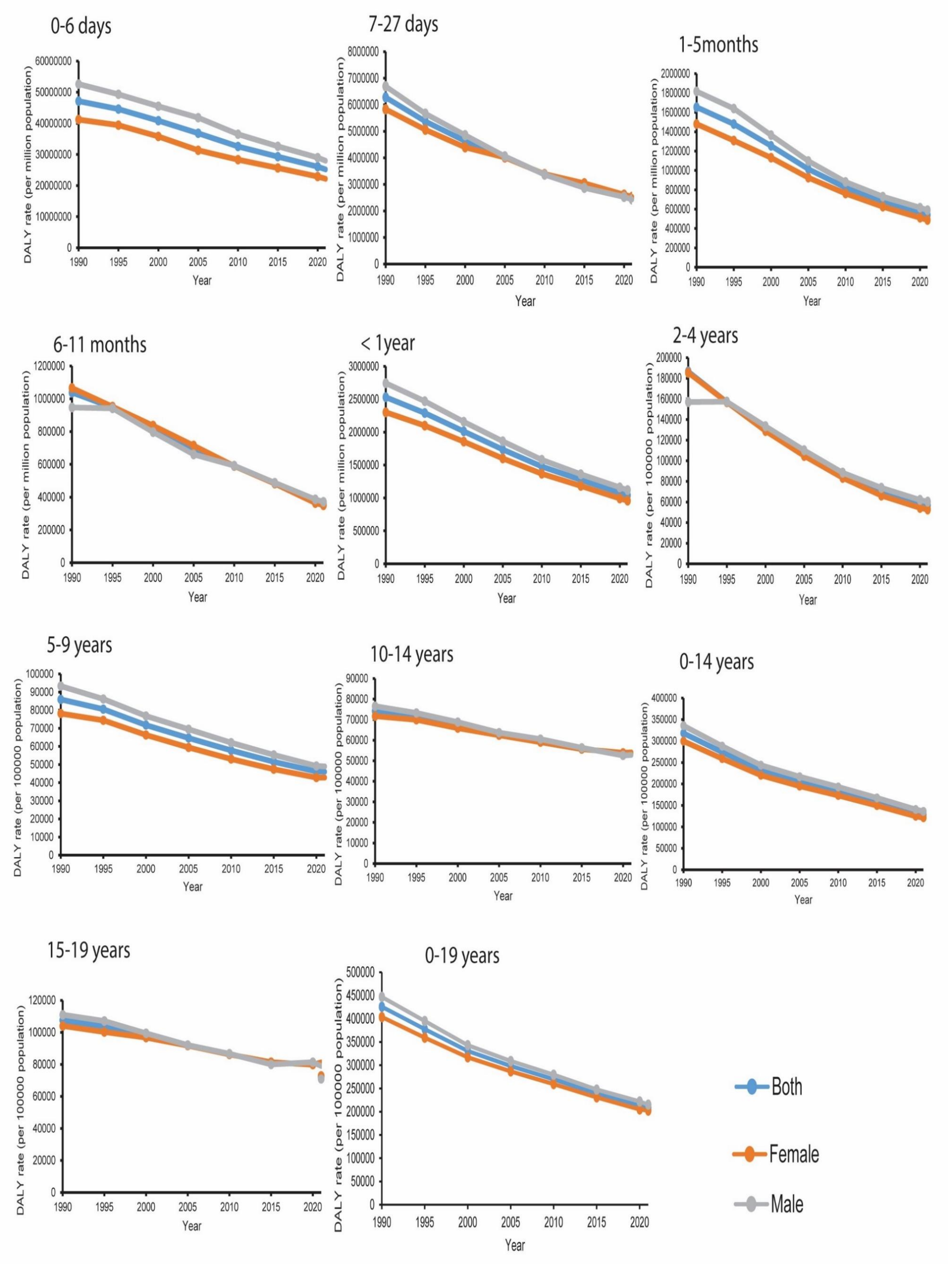

Figure S16. DALY rate per 100000 population due to NCDs among people aged 0-19 years old in the Asia-Pacific region in 2021 by sex.

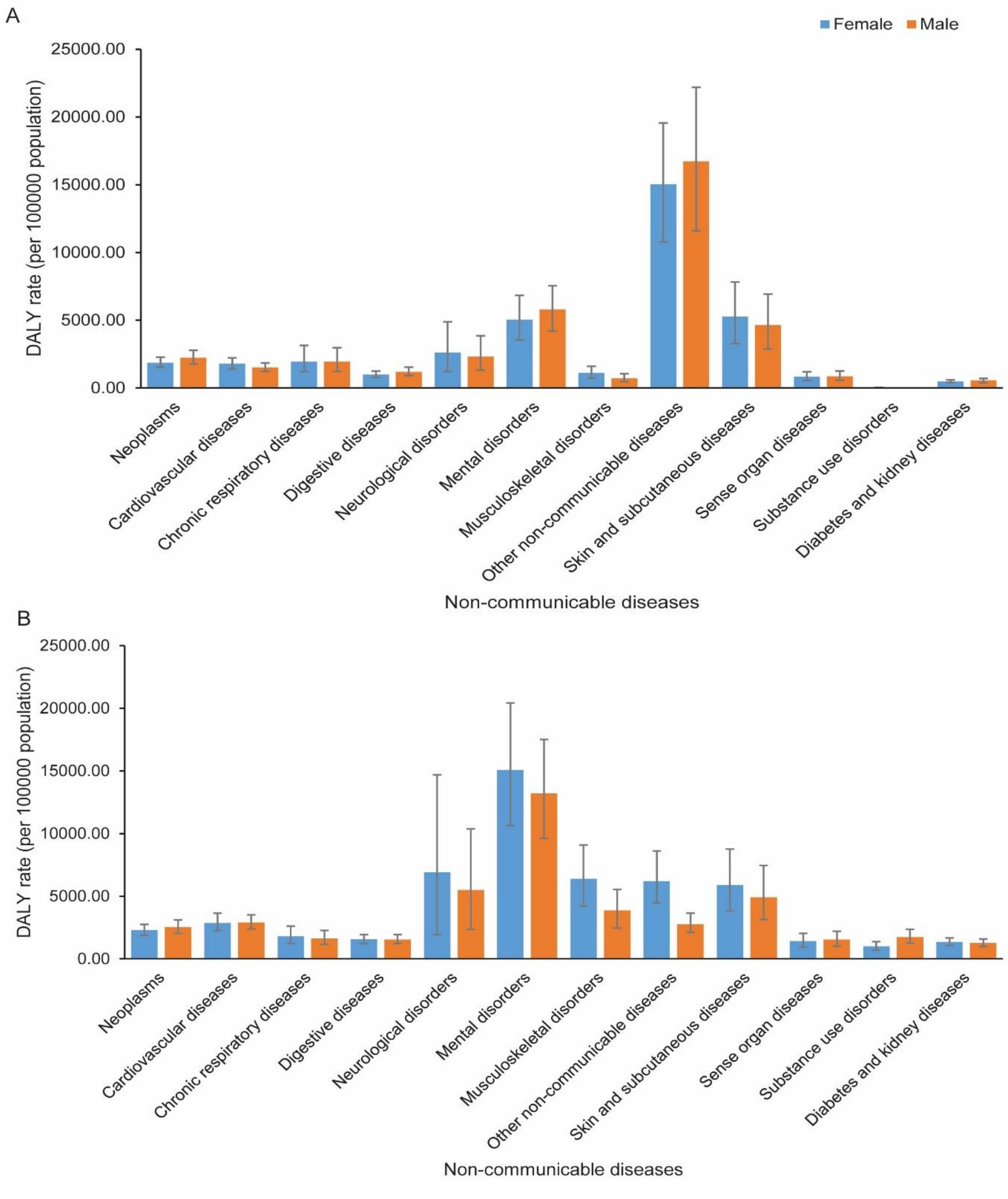

Figure S17. DALY rate per 100000 population due to the first 20 level 3 NCDs among people aged 0-19 years old in the Asia-Pacific region in 2021 by sex.

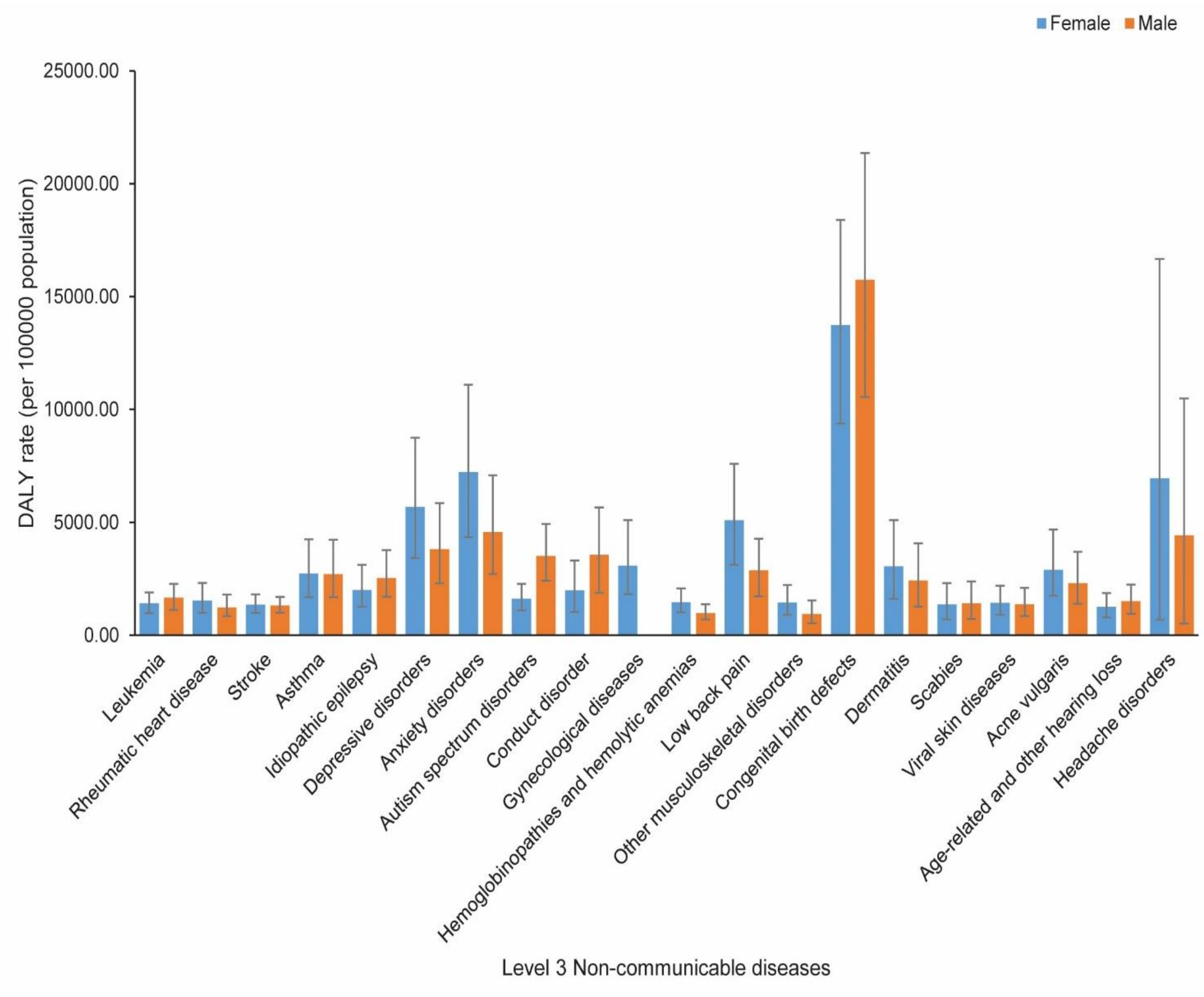

Figure S18. DALY rate per 100000 population due to NCDs among people aged 0-19 years old in the Asia-Pacific region in both sexes from 1990 to 2021.

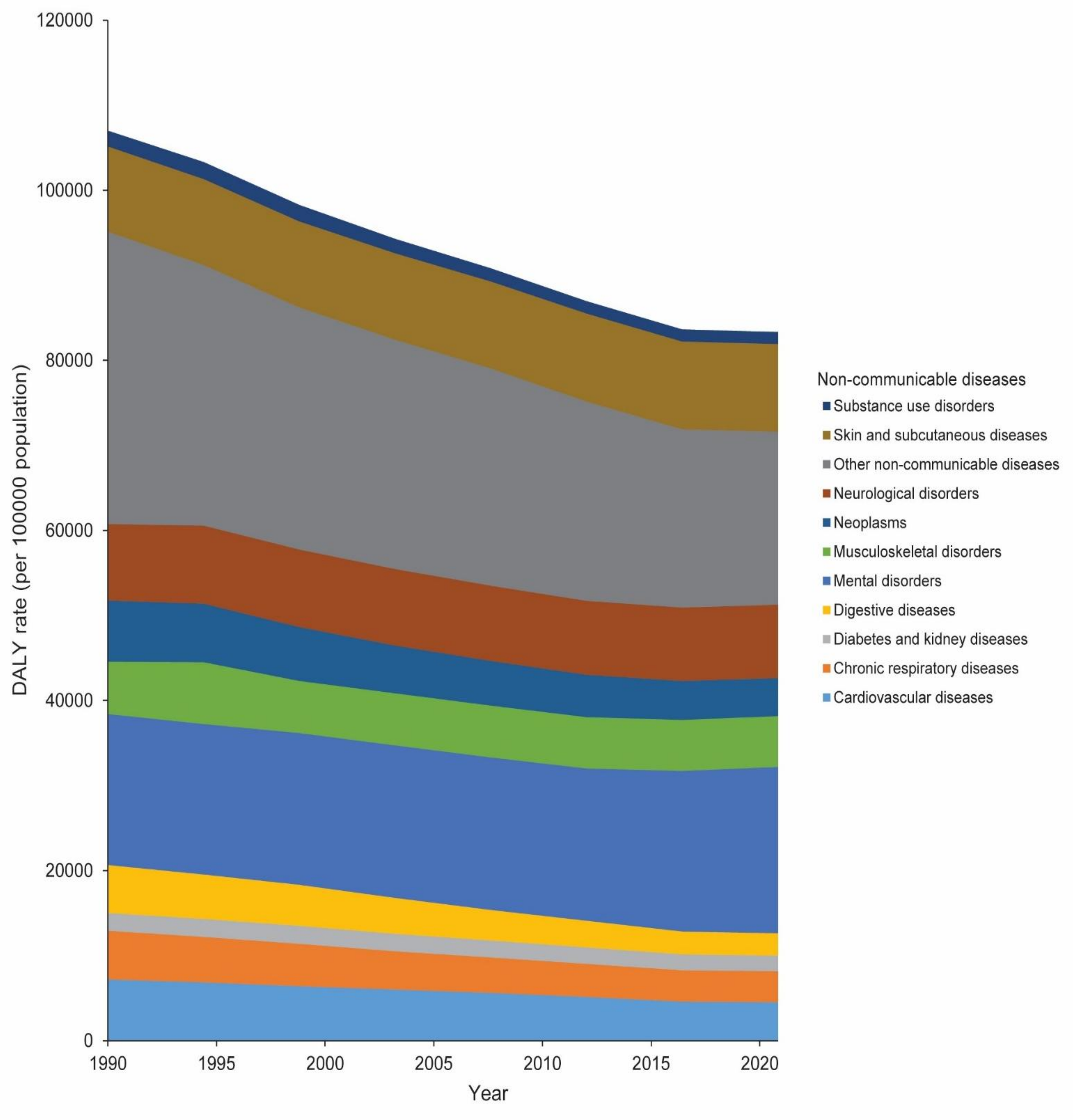

Figure S19. Socio-demographic Index (SDI) in 2021 for countries in the Asia-Pacific region.

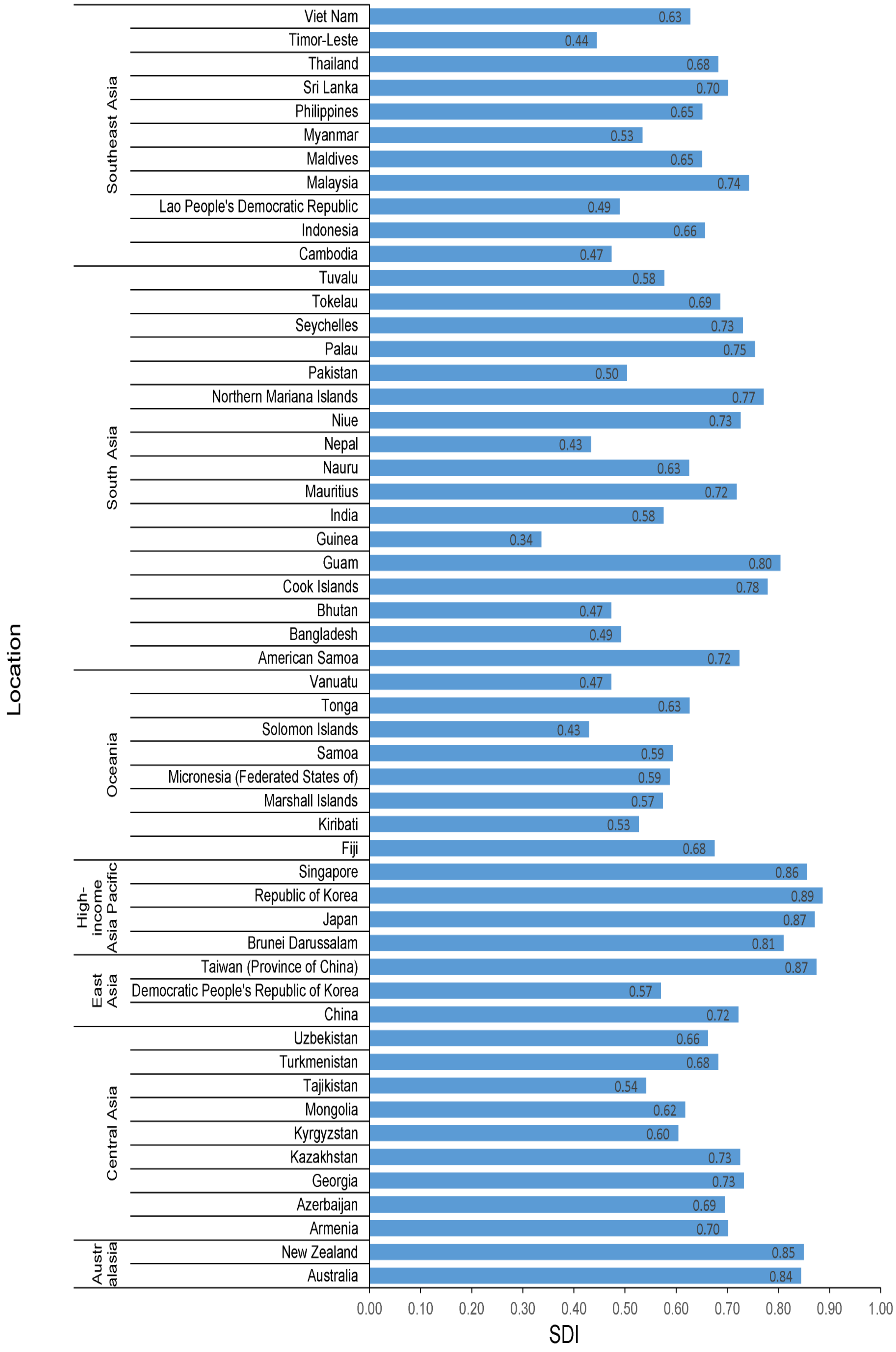

Supplemental research checklist: Revealing compliance with the Guidelines for Accurate and Transparent Health Estimates Reporting (GATHER)

Item #	Checklist item	Reported on page#
**Objectives and funding**		
**1**	Define the indicator(s), populations (including age, sex, and geographic entities), and time period(s) for which estimates were made.	In the method section on pages 5-6 (DALYs, YLLs, and YLDs) and supplemental method.
**2**	List the funding sources for the work.	See the main manuscript on page 20 (in the acknowledgments section).
**Data Inputs**		
For all data inputs from multiple sources that are synthesized as part of the study:		
**3**	Describe how the data were identified and how the data were accessed.	In the method section on pages 5-6 (DALYs, YLLs, and YLDs) and supplemental method.
**4**	Specify the inclusion and exclusion criteria. Identify all ad-hoc exclusions.	In the method section on pages 5-6 (DALYs, YLLs, and YLDs) and supplemental method.
**5**	Provide information on all included data sources and their main characteristics. For each data source used, report reference information or contact name/institution, population represented, data collection method, year(s) of data collection, sex and age range, diagnostic criteria or measurement method, and sample size, as relevant.	In the method section on pages 5-6 (DALYs, YLLs, and YLDs) and supplemental method.<br><br>Data input source tool:<br>https://vizhub.healthdata.org/gbd-results/<br>http://ghdx.healthdata.org/gbd-2021
**6**	Identify and describe any categories of input data that have potentially important biases (e.g., based on characteristics listed in item 5).	In the method section on pages 5-6 (DALYs and YLDs) and supplemental method.<br><br>Capstone GBD 2021 DALYs and YLDs paper. See:<br>https://linkinghub.elsevier.com/retrieve/pii/S0140-6736(24)00757-8<br><br>Capstone GBD 2021 Death paper. See:<br>https://www.thelancet.com/journals/lancet/article/PIIS0140-6736(24)00367-2/fulltext
For data inputs that contribute to the analysis but were not synthesized as part of the study:		
**7**	Describe and give sources for any other data inputs.	Data input source tool:<br>https://vizhub.healthdata.org/gbd-results/<br>http://ghdx.healthdata.org/gbd-2021
For all data inputs:		
**8**	Provide all data inputs in a file format from which data can be efficiently extracted (e.g., a spreadsheet rather than a PDF), including all relevant meta-data listed in item 5. For any data inputs that cannot be shared because of ethical or legal reasons, such as third-party ownership, provide a contact name or the name of the institution that retains the right to the data.	Data input source tool:<br>https://vizhub.healthdata.org/gbd-results/<br>http://ghdx.healthdata.org/gbd-2021
**Data analysis**		
**9**	Provide a conceptual overview of the data analysis method. A diagram may be helpful.	Data analysis section on page 6;<br>Capstone GBD 2021 DALYs and YLDs paper. See:<br>https://linkinghub.elsevier.com/retrieve/pii/S0140-6736(24)00757-8;<br><br>Capstone GBD 2021 Death paper. See:

		https://www.thelancet.com/journals/lancet/article/PIIS0140-6736(24)00367-2/fulltext
**10**	Provide a detailed description of all steps of the analysis, including mathematical formulae. This description should cover, as relevant, data cleaning, data pre-processing, data adjustments and weighting of data sources, and mathematical or statistical model(s).	Capstone GBD 2021 DALYs and YLDs paper. See: https://linkinghub.elsevier.com/retrieve/pii/S0140-6736(24)00757-8<br><br>Capstone GBD 2021 Death paper. See: https://www.thelancet.com/journals/lancet/article/PIIS0140-6736(24)00367-2/fulltext
**11**	Describe how candidate models were evaluated and how the final model(s) were selected.	In the method section on pages 5-6 (DALYs, YLLs, and YLDs), the data analysis section on page 6, and the supplemental method.<br><br>Capstone GBD 2021 DALYs and YLDs paper. See: https://linkinghub.elsevier.com/retrieve/pii/S0140-6736(24)00757-8<br><br>Capstone GBD 2021 Death paper. See: https://www.thelancet.com/journals/lancet/article/PIIS0140-6736(24)00367-2/fulltext
**12**	Provide the results of an evaluation of model performance, if done, as well as the results of any relevant sensitivity analysis.	Capstone GBD 2021 DALYs and YLDs paper. See: https://linkinghub.elsevier.com/retrieve/pii/S0140-6736(24)00757-8<br><br>Capstone GBD 2021 Death paper. See: https://www.thelancet.com/journals/lancet/article/PIIS0140-6736(24)00367-2/fulltext
**13**	Describe methods for calculating uncertainty of the estimates. State which sources of uncertainty were, and were not, accounted for in the uncertainty analysis.	In the method section on pages 5-6 (DALYs, YLLs, and YLDs), the data analysis section on page 6, and the supplemental method.
**14**	State how analytic or statistical source code used to generate estimates can be accessed.	In the method section on pages 5-6 (DALYs, YLLs, and YLDs), the data analysis section on page 6, and the supplemental method.<br><br>Data input source tool:<br>https://vizhub.healthdata.org/gbd-results/<br>http://ghdx.healthdata.org/gbd-2021
**Results and Discussion**		
**15**	Provide published estimates in a file format from which data can be efficiently extracted.	In the result section on pages 7-16 and supplemental Tables and Figures.
**16**	Report a quantitative measure of the uncertainty of the estimates (e.g. uncertainty intervals).	In the result section on pages 7-16.
**17**	Interpret results in light of existing evidence. If updating a previous set of estimates, describe the reasons for changes in estimates.	In the discussion section on pages 16-18.
**18**	Discuss limitations of the estimates. Include a discussion of any modelling assumptions or data limitations that affect interpretation of the estimates.	In the discussion section pages 18-19.

References

1. **Global incidence, prevalence, years lived with disability (YLDs), disability-adjusted life-years (DALYs), and healthy life expectancy (HALE) for 371 diseases and injuries in 204 countries and territories and 811 subnational locations, 1990-2021: a systematic analysis for the Global Burden of Disease Study 2021**. *Lancet* 2024, **403**(10440):2133-2161.
2. Stevens GA, Alkema L, Black RE, Boerma JT, Collins GS, Ezzati M, Grove JT, Hogan DR, Hogan MC, Horton R *et al*: **Guidelines for Accurate and Transparent Health Estimates Reporting: the GATHER statement**. *The Lancet* 2016, **388**(10062):e19-e23.
3. **Global burden of 288 causes of death and life expectancy decomposition in 204 countries and territories and 811 subnational locations, 1990-2021: a systematic analysis for the Global Burden of Disease Study 2021**. *Lancet* 2024, **403**(10440):2100-2132.
